# Activation of the osteoblastic HIF-1α pathway partially alleviates the symptoms of STZ-induced type 1 diabetes mellitus via RegIIIγ

**DOI:** 10.1038/s12276-024-01257-4

**Published:** 2024-07-01

**Authors:** Minglong Qiu, Leilei Chang, Guoqing Tang, Wenkai Ye, Yiming Xu, Nijiati Tulufu, Zhou Dan, Jin Qi, Lianfu Deng, Changwei Li

**Affiliations:** 1 Department of Orthopedics, Shanghai Key Laboratory for Prevention and Treatment of Bone and Joint Diseases, Shanghai Institute of Traumatology and Orthopedics, Ruijin Hospital, Shanghai Jiao Tong University School of Medicine, 197 Ruijin 2nd Road, Shanghai, 200025 China; 2 Kunshan Hospital of Traditional Chinese Medicine, Affiliated Hospital of Yangzhou University, 388 Zuchongzhi Road, Kunshan, 215300 Jiangsu China

**Keywords:** Bone development, Translational research, Type 1 diabetes, Osteoporosis

## Abstract

The hypoxia-inducible factor-1α (HIF-1α) pathway coordinates skeletal bone homeostasis and endocrine functions. Activation of the HIF-1α pathway increases glucose uptake by osteoblasts, which reduces blood glucose levels. However, it is unclear whether activating the HIF-1α pathway in osteoblasts can help normalize glucose metabolism under diabetic conditions through its endocrine function. In addition to increasing bone mass and reducing blood glucose levels, activating the HIF-1α pathway by specifically knocking out *Von Hippel‒Lindau* (*Vhl*) in osteoblasts partially alleviated the symptoms of streptozotocin (STZ)-induced type 1 diabetes mellitus (T1DM), including increased glucose clearance in the diabetic state, protection of pancreatic β cell from STZ-induced apoptosis, promotion of pancreatic β cell proliferation, and stimulation of insulin secretion. Further screening of bone-derived factors revealed that islet regeneration-derived protein III gamma (RegIIIγ) is an osteoblast-derived hypoxia-sensing factor critical for protection against STZ-induced T1DM. In addition, we found that iminodiacetic acid deferoxamine (SF-DFO), a compound that mimics hypoxia and targets bone tissue, can alleviate symptoms of STZ-induced T1DM by activating the HIF-1α-RegIIIγ pathway in the skeleton. These data suggest that the osteoblastic HIF-1α-RegIIIγ pathway is a potential target for treating T1DM.

## Bone hormone link to diabetes: HIF-1α pathway activation

The skeleton isn’t just for support, it also helps control body functions. This research looked at how a specific process in bone-forming cells, called the hypoxia-inducible factor-1 alpha (HIF-1α) pathway, affects sugar breakdown and diabetes. The scientists discovered that triggering this process in these cells can help manage sugar levels in diabetes through a protein named RegIIIγ. They also found that a substance named SF-DFO, which imitates low oxygen conditions and focuses on bone tissue, can somewhat ease type 1 diabetes symptoms by triggering the HIF-1α-RegIIIγ process in the skeleton. This implies that this specific process in bone-forming cells could be a possible treatment for type 1 diabetes.

This summary was initially drafted using artificial intelligence, then revised and fact-checked by the author.

## Introduction

The skeleton provides crucial structural support for the body, enabling movement and protecting internal organs^1^. Furthermore, over the past decade, the skeleton has been recognized as a critical endocrine organ that regulates various bodily functions, maintaining the normal metabolic activities of multiple organs by releasing various bone-derived factors^2–5^. For example, osteocalcin produced by osteoblasts plays a critical role in promoting pancreatic β-cell proliferation, stimulating insulin secretion, increasing insulin sensitivity, and regulating male fertility^6–8^. In contrast, osteocytes can regulate phosphorus and peripheral fat metabolism by secreting fibroblast growth factor 23 and sclerostin^9–12^. Therefore, any dysregulation of bone homeostasis can lead to systemic regulatory dysfunction. One extreme case is the elimination of osteoblasts, which can cause severe hematopoietic abnormalities, abnormal immune regulation, and disruptions in glucose metabolism^13–15^. Thus, proper skeletal growth and development, as well as the maintenance of bone homeostasis, are crucial for the overall health of an organism.

Bone cells, especially osteoblasts, are oxygen-sensing cells that grow and differentiate under low-oxygen conditions^16–21^. HIFs are the primary regulators of the adaptive response to alterations in oxygen tension^22,23^. Studies have shown that proteins related to the oxygen-sensing pathway, such as von Hippel‒Lindau tumor suppressor protein (VHL), prolyl hydroxylase-domain proteins (PHDs), and hypoxia-inducible factors (HIFs), are expressed in osteoblasts^21,24^. The inactivation of HIF-1α in osteoblasts significantly reduced trabecular bone volume, decreases the rate of bone formation, and modifies the architecture of cortical bone^20,21^. Conversely, the deletion of PHD or VHL stabilizes HIF-1α, increasing bone mass by regulating osteoblast bone formation and osteoclast bone resorption^25,26^. Therefore, the important role of the HIF-1α pathway in regulating bone homeostasis indicates that this molecule is an excellent target for investigating the role of bone cells in controlling the normal functioning of organs in distant regions of the body through its endocrine function.

In addition to maintaining normal bone homeostasis, the HIF-1α pathway in osteoblasts regulates various metabolic processes in the body. For example, activation of HIF signaling in osteoblast lineage cells promotes remote breast cancer cell growth and dissemination, including in the lungs and other peripheral tissues, by increasing chemokine (C-X-C motif) ligand 12 expression and circulation in the blood^27^. Furthermore, activation of the HIF-1α signaling pathway in osteoblasts facilitates erythropoietin secretion to regulate the hematopoietic microenvironment^24^. In addition, as a critical molecule in the conversion of aerobic metabolism and glycolytic processes, HIF-1α activation, specifically in bone lineage cells due to *Vhl* deletion, increased glucose glycolysis and utilization, which led to an overall increase in glucose uptake from the circulation by the skeleton that was correlated with reduced blood glucose levels^28^. In addition to hypoglycemia, another common symptom of abnormal glucose metabolism is hyperglycemic diabetes, which is mainly characterized by high blood glucose, pancreatic damage, and insulin resistance. However, it is unclear whether activating the HIF-1α pathway in osteoblasts can help normalize glucose metabolism under diabetic conditions through its endocrine function.

In addition to increasing bone mass and reducing blood glucose levels, this study revealed that activating the HIF-1α pathway by specifically knocking out *Vhl* in osteoblasts partially alleviated the symptoms of streptozotocin (STZ)-induced type 1 diabetes mellitus (T1DM). Further screening of bone-derived factors revealed that islet regeneration-derived protein III gamma (RegIIIγ) is an osteoblast-derived hypoxia-sensing factor that plays a critical role in protecting against STZ-induced T1DM. In addition, iminodiacetic acid deferoxamine (SF-DFO), a compound that mimics hypoxia and targets bone tissue, can partially alleviate symptoms of STZ-induced T1DM by activating the HIF-1α-RegIIIγ pathway in the skeleton. Thus, our data suggest that the osteoblastic HIF-1α-RegIIIγ pathway is a potential target for treating T1DM.

## Materials and methods

### Mice

#### Generation of *Vhl* conditional knockout mice

*Vhl*^*flox/flox*^ C57BL/6 breeding pairs were kindly provided by Dr. Thomas L. Clemens (Department of Orthopedic Surgery, John Hopkins University School of Medicine, Baltimore, MD). *Ocn-Cre* C57BL/6 breeding pairs (stock no: T001875) were purchased from GemPharmatech Co., Ltd. (Nanjing, Jiangsu Province, China). *Dmp-1-Cre* mice were purchased from the Jackson Laboratory (FVB/N, JAX Stock #023047; Sacramento, CA) and backcrossed to mice with a C57/BL6J background over at least four generations to ensure that all mice were maintained with the C57BL/6 background. *Vhl* cKO mice (*Vhl*^*flox/flox*^*; Ocn-Cre*^*+/−*^) and their littermate controls (*Vhl*^*flox/flox*^*; Ocn-Cre*^*−/−*^) were obtained by the crossbreeding of *Vhl*^*flox/flox*^ and Ocn-Cre mice. *Vhl*^*flox/flox*^*; Dmp-1-Cre*^*+/−*^ mice and their littermate controls (*Vhl*^*flox/flox*^*; Dmp-Cre*^*−/−*^) were obtained by the crossbreeding of *Vhl*^*flox/flox*^ and Dmp-1-Cre mice. Genotypes were determined by PCR amplification of purified tail genomic DNA using primers for Supplementary Tables 1 and 2.

#### Generation of *RegIIIγ* conditional knockout mice

*RegIIIγ* conditional knockout mice were generated by GemPharmatech Co., Ltd. (Nanjing, China). The mice were generated via the CRISPR/Cas9 system. First, two sgRNAs targeting introns on both sides of the floxed region of the RegIIIγ gene were constructed and transcribed in vitro. Moreover, a donor vector with a loxP fragment was designed and constructed in vitro. Then, Cas9 mRNA, sgRNA, and the donor were coinjected into zygotes. After that, the zygotes were transferred into the oviduct of pseudopregnant ICR females at 0.5 DPC. F0 mice were birthed after 19 ~ 21 days of transplantation, and PCR and sequencing of tail DNA were used to identify all the offspring of the ICR females (F0 mice). These methods were used to genotype F0 mice. Finally, we crossed F0 mice with C57BL/6J mice to generate heterozygous mice. *RegIIIγ* cKO mice (*RegIIIγ*^*flox/ flox*^*; Ocn-Cre*^*+/−*^) and their littermate controls (*RegIIIγ*^*flox/flox*^*; Ocn-Cre*^*−/−*^) were obtained by the crossbreeding of *RegIIIγ*^*flox/flox*^ and Ocn-Cre mice. Genotypes were determined by PCR amplification of purified tail genomic DNA using the primers in Supplementary Tables 2 and 3.

#### Generation of *Vhl* and *RegIIIγ* double conditional knockout mice

Mice with osteoblastic *Vhl* and *RegIIIγ* conditional deletions were generated by crossing *Vhlf*^*lox/flox*^-Ocn-Cre^+/−^ mice (*Vhl* cKO mice) with *RegIIIγ*^*flox/flox*^-Ocn-Cre^+/−^ mice (*RegIIIγ* cKO mice) to generate *Vhlf*^*lox/flox*^-*RegIIIγ*^*flox/flox*^-Ocn-Cre^+/−^ mice (referred to as *Vhl-RegIIIγ* cKO mice). For the control group, *Vhlf*^*lox/flox*^-*RegIIIγ*^*flox/flox*^-Ocn-Cre^−/−^ littermates were used (referred to as *Vhl*-*RegIIIγ*^*flox/flox*^ mice). Genotypes were determined by PCR amplification of purified tail genomic DNA using the primers listed in Supplementary Tables 1–3.

C57BL/6 mice were purchased from Weitong Lihua Laboratory Animal Technology Co. (Shanghai, China). The animals were housed with free access to water and food in an air-conditioned room with a 12-h light‒dark cycle at 21–23 °C and 60% relative humidity in the animal facility at Ruijin Hospital, Shanghai Jiao Tong University School of Medicine, Shanghai, China. For all mouse studies, preliminary experiments were conducted to determine the requirements for sample size.

### STZ-induced islet injury

Mice were fasted for 6 h prior to STZ injection. STZ (Sigma-Aldrich, V900890) was dissolved in citrate buffer (Sigma-Aldrich, C2488) to generate the injection solution. Mice were given intraperitoneal injections of 40 mg of STZ per kg of body weight once daily for 5 days. Aged mice were injected with the same dose for 3 days. The control group was injected with the same volume of citrate buffer.

### RegIIIγ neutralization in vivo

A RegIIIγ neutralizing antibody was used to block its function, as confirmed in our previous reports^29^. A dose of 10 mg/kg RegIIIγ-neutralizing antibody was administered to male *Vhl* cKO mice through the tail vein 24 h before the initial STZ intraperitoneal injection. Subsequently, a daily dose of 10 mg/kg RegIIIγ-neutralizing antibody was administered through the tail vein after each subsequent intraperitoneal STZ injection, for a total of 5 consecutive days.

### Islet isolation

Islet isolation was conducted by first anesthetizing the male mice with pentobarbital sodium at a dose of 40 mg/kg. The common bile duct and Vater’s ampulla were then ligated. A 32G syringe needle was used to inject 3 ml of collagenase V solution (C9263, Sigma, with a concentration of 0.8 mg ml^−1^ in D-Hank’s buffer) into the common bile duct. The pancreas was subsequently removed and placed in 5 ml of collagenase V solution for 15 min at 37 °C. After digestion, 40 ml of prechilled Hanks’ buffer and 5% BSA were added to halt the digestion process. The pancreas was thoroughly dispersed using a vortex mixer and then filtered through a 400 μm mesh filter. Next, the mixture was allowed to stand on ice for 10 min before 30 ml of the supernatant was removed. Then mixed with 30 ml of prechilled Hanks’ buffer. This step was repeated 4 times. The final pellet was obtained by centrifugation at 500 × *g* for 2 min. Islets were manually selected under a stereomicroscope. The isolated islets were cultured in RPMI-1640 medium supplemented with 10% fetal bovine serum and 10% penicillin‒streptomycin for islet identification, counting, and glucose-stimulated insulin secretion experiments.

### Islet equivalent calculation

The islet equivalent (IEQ) calculation was performed as previously reported^30^. Isolated islets were cultured for 24 h and then stained with dithizone (DTZ) (Sigma) solution for 10 min (50 μg/ml). The method for preparing the DTZ solution was previously described^31^. In summary, 50 mg of DTZ was dissolved in 5 ml of DMSO to create a stock solution. For use, 1 ml of the stock solution was dissolved in Hanks buffer containing 5% FBS. After staining, the medium was replaced, and images were captured using a Zeiss microscope (Germany). As described previously^30^, the long and short diameters as well as the circumference of each islet were measured using ImageJ. The total volume of all islets was then calculated using a diameter of 150 μm as the islet equivalent. Finally, the total islet equivalent of each mouse was calculated.

### Glucose-stimulated insulin secretion

Intraperitoneal glucose-stimulated insulin secretion (GSIS) was performed as previously reported^28^. Mice were fasted for 8 h. Prior to glucose injection, blood samples were taken from the tail tip to establish baseline insulin levels. Then, a glucose solution (2 g/kg) was injected intraperitoneally, and blood samples were collected from the tail tip at 5, 10, and 15 min after the injection. After centrifugation, the serum insulin levels were measured using the method specified above.

For in vitro GSIS, following a previously described procedure^32^, isolated pancreatic islets were cultured for 24 h and then transferred to a 24-well culture plate. After the cells were stimulated with 1 mmol/l STZ and 50 ng/ml or 200 ng/ml RegIIIγ for 12 h, they were preincubated for 1 h in KRBH buffer containing 0.1% BSA and 2 mM glucose and subsequently switched to KRBH buffer supplemented with 2.8 mM glucose (low glucose) for another hour. Afterward, the islets were incubated in KRBH buffer containing 16.7 mM glucose (high glucose) for 1 h. The culture supernatants from both the low- and high-glucose conditions were collected, and insulin levels were measured following the methods described above.

### SF-DFO treatment

The method for the synthesis of SF-DFO was described in our previous study^33^. SF-DFO was dissolved in PBS (Sigma-Aldrich, TMS-012-A) to prepare the injection solution. SF-DFO was administered intraperitoneally to male mice at 50 mg/kg body weight once every other day for 1 month. The control group was injected with the same volume of PBS. For determination of the effect of SF-DFO on islets, islet injury was induced at the end of the SF-DFO course, as previously described.

### Metabolic studies and bioassays

For the GTT, glucose was administered intraperitoneally at 2 g/kg body weight after an overnight fast. Blood glucose was measured using a Roche^®^ Excellence Gold Collection^®^ monitor (ACCU-CHEK) at 5, 15, 30, 60, and 120 min post-injection. ELISAs were used to determine the serum insulin (Invitrogen, EMINS), RegIIIγ (Cusabio, CSB-EL019549MO), LCM2 (R&D, MLCN20), and OCN (Abcam, ab285236) levels.

### Cell culture

Osteoblasts were extracted from 3-day-old neonatal *Vhl*^*flox/flox*^ mouse calvariae^34^. After removal of the epidermis and muscle from the calvaria, the calvaria was digested for 10 min at 37 °C in a water bath using 80 mg/ml collagenase type I (Gibco, 17100017) to remove the digestive fluid. This step was repeated once. After removal of the digest, the calvariae were sheared and digested with 80 mg/ml collagenase type I for 20 min, after which the digest was centrifuged at 1500 rpm for 5 min to collect the centrifuged cells. This procedure was repeated three times. The cells obtained three times were mixed together. Cells were seeded on 60 × 15 mm style culture dishes (Corning, 430166) and cultured in α-MEM (Gibco, 12571048) supplemented with 100 IU/ml penicillin, 100 mg/ml streptomycin (Gibco, 15140122), and 10% fetal bovine serum (Gibco, 10099141). After the second generation, the extracted osteoblasts were used for subsequent experiments.

### Hypoxic culture of bone tissue

Male mice were euthanized using CO_2_ to obtain femurs and tibias. Fibroblasts were removed by digestion with 80 mg/ml collagenase type I (Gibco, 17100017) at 37 °C for 20 min. The bone marrow was washed with PBS and incubated in a hypoxic incubator for 24 h for subsequent experiments. Bone tissues were cultured in 60 × 15 mm style culture dishes (Corning, 430166) and cultured in α-MEM (Gibco, 12571048) supplemented with 100 IU/ml penicillin, 100 mg/ml streptomycin (Gibco, 15140122), and 10% fetal bovine serum (Gibco, 10099141).

### Adenovirus infection

The extracted osteoblasts were digested with 0.25% trypsin-EDTA (Gibco, 25200072) and inoculated at 3 × 10^5^ cells/ml in 24-well culture plates (Corning, 353047). The cells were incubated at 37 °C and 5% CO_2_ overnight. Before infection, the adenovirus was slowly melted on ice, the original medium was aspirated, a 1/2 volume of fresh medium was added, and Cre-adenovirus with GFP (Hanbio Biotechnology, Shanghai, China) was added and mixed gently. The control adenovirus with GFP was added as a control. After 4 h of infection, the volume was replenished to the complete culture volume. At 10–16 h post-infection, the virus-containing culture medium was discarded, the medium was replaced with fresh complete culture medium, and the cells were incubated at 37 °C. GFP expression efficiency was observed by fluorescence microscopy 24–48 h post-infection. Pre-experiments were used to determine the multiplicity of infection before the formal experiment.

### Quantitative real-time PCR

TRIzol reagent (Invitrogen, 15596026) was used for total RNA extraction. For reverse transcription of mRNA, 1000 ng of total RNA was reverse transcribed with PrimeScript^TM^ RT Master Mix (TaKaRa, RR036A). Quantitative real-time PCR was performed to amplify cDNA with TB Green^®^ Fast qPCR Mix (TaKaRa, RR430A) and an ABI 7500 Sequencing Detection System (Applied Biosystems, USA). *β-Actin* was used as an endogenous control for the quantitation of mRNA expression. The specific sequences of primers used for real-time RT‒PCR are listed in Supplementary Table 4 in the Supplementary Materials.

### Western blot

Cell samples were lysed on ice in RIPA buffer (Beyotime, P0013B) for 20 min. The tissue samples were ground to powder in a mortar containing liquid nitrogen before lysis using RIPA buffer. Lysed samples were collected and centrifuged at 14,000 × *g* for 10 min to remove cellular and tissue debris. The protein concentration was determined using a BCA Protein Assay Kit (Beyotime, P0009). Each sample containing 10 μg of total protein was separated by 10% SDS‒PAGE (Beyotime, P0690) and transferred to a PVDF membrane. Proteins with molecular weights less than 20 kDa were transferred to 0.2 µm PVDF membranes (Thermo Scientific, 88520), and proteins with molecular weights greater than 20 kDa were transferred to 0.45 µm PVDF membranes (Thermo Scientific, 88518). Nonspecifically bound proteins were blocked with Western Quick-Block^TM^ (Beyotime, P0252) for 10 min, and the membranes were incubated with primary antibodies against actin (Abcam, ab179467), HIF-1α (Abcam, ab179483), and RegIIIγ (Abcam, ab198216) at 4 °C overnight. After three washes with TBST, the membranes were incubated with horseradish peroxidase-conjugated secondary antibodies. The antibody-antigen complexes were visualized with Immobilon reagent (Millipore, P90719).

### Histomorphometry

Bone tissue was decalcified and then embedded in paraffin. Pancreatic tissue was fixed using 4% paraformaldehyde (Beyotime, P0099) and then embedded in paraffin. Five-micron-thick sections were stained with H&E and Goldner stains according to standard methods. The bone area/tissue area was calculated using ImageJ; one section per sample and three samples were analyzed. The pancreatic islet number and pancreatic area were calculated using ImageJ; one section per sample and five samples were analyzed.

### Immunohistochemistry/immunocytochemistry

For IHC analysis, deparaffinized sections were heated in sodium citrate buffer (Beyotime, P0083) for ~20 min at 95–100 °C. The sections were incubated with 3% H_2_O_2_ for 15 min and then treated with 5% BSA for 30 min. Next, the sections were incubated with primary antibodies against PCNA (Abcam, ab29), BAX (Abcam, ab32503), insulin (CST, 3014), HIF-1α (Abcam, ab114977), and RegIIIγ (Abcam, ab198216) overnight at 4 °C. The cells were then incubated with biotin-conjugated secondary antibodies and visualized using a streptavidin-biotin staining technique. Nuclei were stained with hematoxylin, and slides were photographed using a microscope (Zeiss). For all staining analyses, samples understained due to technical problems were excluded. For IF analysis, cultured cells were fixed using 4% paraformaldehyde at room temperature for 15 min for subsequent manipulation. Tissue sections were deparaffinized for subsequent manipulation. The samples were treated with PBS buffer containing 5% BSA and 0.3% Triton X-100 for 60 min. The samples were incubated overnight at 4 °C with insulin (CST, 3014) and RegIIIγ (ABclonal, A2146) primary antibodies. The specimens were incubated in the dark for 1–2 h at room temperature with fluorochrome-conjugated secondary antibodies diluted in antibody diluent. The samples were then incubated for 10 min with DAPI and photographed using a laser scanning confocal microscope (Zeiss).

### Microcomputed tomography

Micro-CT analysis was performed on the left femur of each mouse, as described previously^35^. After fixation with 4% paraformaldehyde, the femur was scanned on a Skyscan 1172 (Aartselaar, Belgium) with an isotropic voxel size of 10 μm, 50 keV, 500 μA and a rotation step of 0.7^36^. The trabecular bone parameters, including bone mineral density (BMD, g/cm3), BV/TV, trabecular thickness (Tb.Th, mm), and trabecular separation (Tb.Sp, mm), in the distal metaphysis of the femurs were measured as previously reported^37,38^. We began analyzing slices at the bottom of the distal growth plate, where the epiphyseal cap structure had completely disappeared, and continued analyzing 200 slices (5 μm/slice) toward the proximal end of the femur. Therefore, in this study, the region of interest (ROI) of the bone trabeculae extended 1 mm from the end of the distal femoral growth plate to the proximal end.

### Luciferase reporter assay

The −2000 → −1 bp region of the *RegIIIγ* encoding region was selected as the promoter region for analysis. This promoter region was then analyzed using the JASPAR core database, which revealed the presence of two putative hypoxia response elements (HRE) at −951 → −954 bp (sense strand of DNA) and −812 → −815 bp (antisense strand of DNA) bp relative to the transcription start site on the *RegIIIγ* promoter. The specific sequence for these HREs was RCGTG, where R is either A or G. The *RegIIIγ* promoter reporter was directly cloned and inserted into a pGL3-Basic luciferase vector using the primers listed in Supplementary Table 5 to amplify the mouse RegIIIγ promoter via PCR. Mouse primary osteoblasts were then seeded into 24-well plates and cotransfected with different plasmids, including firefly reporter constructs containing the *RegIIIγ* promoter, a Renilla-expressing plasmid, and varying doses of the HIF-1α plasmid. Firefly and Renilla luciferase activities were measured 24 h post-transfection using a Dual-Luciferase Assay System (Promega).

### ChIP‒qPCR

The ChIP procedure was performed as previously described^39^ utilizing a ChIP assay kit (Millipore Sigma, Burlington, MA). Briefly, the cells were fixed with formaldehyde, and 125 mM glycine was used to neutralize the reaction. The cells were lysed with a mixture of 5 mM PIPES (pH 8), 85 mM KCl, 0.5% NP-40, 20 mM sodium butyrate, and protease/phosphatase inhibitors (2 mM PMSF, 20 mM NaF, 1X aprotinin, 0.1 mg/ml leupeptin, and 2 mM Na_3_VO_4_). Chromatin fragments of 100–500 base pairs were obtained through sonication with a Bioruptor sonicator (Diagenode, Denville, NJ). With 3 μg of HIF-1α antibody (Novus, NB100-105) or negative control anti-IgG (CST, #5415), fragmented chromatin (200 μg) was incubated overnight to form immune complexes, which were coupled to Protein A beads. Crosslinks were reversed (65 °C for 12–16 h), and then, the precipitated DNA was purified using the QIAquick PCR purification kit (Qiagen, Valencia, CA) after being treated with proteinase K and RNase A. The input and purified HIF-1α-ChIP-DNA were used for qPCR. The values obtained from the immunoprecipitated samples were normalized to those from the input DNA. The DNA-star Lasergene 15.2 core suite (DNA-star, Madison, WI) was used to design primers for the putative HIF-1α-binding regions −951 → −954 (sense strand of DNA) and −812 → −815 (antisense strand of DNA) on the *RegIIIγ* promoter. All primers were designed through Primer-BLAST, and primer sequences are included in Supplementary Table 6. Immunoprecipitated DNA was analyzed through RT‒qPCR as previously described. With the ^ΔΔ^Ct method and normalization to % input, fold changes were calculated and are presented as fold enrichment relative to the control mouse IgG.

### Whole-transcriptome analysis

#### RNA isolation and library preparation

Long bones (femora and tibia) were dissected from 8-week-old male *Vhl* cKO mice (Ocn-Cre;Vhl^f/f^) and their littermate controls (Vhl^f/f^), with 4 mice in each group. The ends of the femora and tibia were cut open, leaving only the bone shaft. The bone marrow was then flushed with PBS using a syringe. The bone was minced and homogenized using FastPrep-24™ 5G (MP Biochemicals). The homogenized tissue was resuspended in TRIzol reagent (Sigma-Aldrich, T9424). RNA was isolated following the protocol provided by the manufacturer. The purity and quantity of the RNA were assessed using a NanoDrop 2000 spectrophotometer (Thermo Scientific, USA). The integrity of the RNA was evaluated using an Agilent 2100 Bioanalyzer (Agilent Technologies, Santa Clara, CA, USA). The libraries were constructed using the VAHTS Universal V6 RNA-seq Library Prep Kit following the instructions provided by the manufacturer. Transcriptome sequencing and analysis were performed by OE Biotech Co., Ltd. (Shanghai, China).

#### RNA sequencing and differentially expressed gene analysis

The libraries were sequenced using an Illumina NovaSeq 6000 platform, generating 150 bp paired-end reads. Approximately 6 Gb of raw reads were generated for each sample. The raw reads, in fastq^40^ format, were first processed using fastp to remove low-quality reads and obtain clean reads. Approximately 4 Gb of clean reads were retained for each sample for subsequent analyses. The clean reads were mapped to the house mouse genome using HISAT2^41^. The FPKM^42^ values for each gene were calculated, and the read counts for each gene were obtained using HTSeq-count^43^. PCA was performed in R (v 3.2.0) to evaluate the biological duplication of samples.

Differential expression analysis was conducted using DESeq2^43^. A *p* value of <0.05 and a fold change of >1.5 or <0.66 were set as thresholds for significantly differentially expressed genes (DEGs). The raw data were submitted to the NCBI BioProject database under accession number PRJNA1071292. Hierarchical cluster analysis of DEGs was performed in R (v 3.2.0) to demonstrate the expression patterns of genes in different groups and samples. A radar map of the top 30 genes was drawn to show the expression of upregulated or downregulated DEGs using the R package “gradar”.

GO^44^, KEGG^45^ pathway, Reactome, and WikiPathways enrichment analyses of the DEGs were performed using R (v 3.2.0), and the hypergeometric distribution was determined. Significantly enriched terms were screened. Column diagrams, chord diagrams, and bubble diagrams of the significant enrichment terms were drawn using R (v 3.2.0).

### Cytokine array

Blood was collected from the eyes of 8-week-old male *Vhl* CKO (Ocn-Cre;Vhl^f/f^) mice and their littermate controls (Vhl^f/f^). A pipette was used to collect 500 µl of blood from each mouse. The collected blood samples were placed in a serum separator tube and allowed to clot for 2 h at room temperature. After clotting, the samples were centrifuged for 20 min at ~2000 × *g*. The supernatants, which contained the serum, were extracted for cytokine evaluation. Each group consisted of 4 mice, resulting in 4 independent serum samples for each group. Cytokine arrays were performed using the Proteome Profiler Mouse XL Cytokine Array Kit (Cat. # ARY028, R&D Systems) following the manufacturer’s protocol as previously reported^46^. This assay can detect 111 different cytokines, growth factors, and other mediators, allowing semiquantitative analysis. The assay uses a membrane-based sandwich immunoarray with duplicate spots of captured antibodies on a nitrocellulose membrane. The target proteins present in the sample bind to the capture antibodies and are detected using biotinylated detection antibodies. The proteins were visualized using chemiluminescent detection reagents (Bio-Rad) and imaged using a Tanon-5200 Chemiluminescent Imaging System (Tanon Science and Technology) (Supplementary Fig. 7). For analysis, the chemiluminescence signals were detected and quantified using a microarray imager and ImageJ software (NIH, Bethesda, MD). The raw data are shown in Supplementary Table 7. The pixel density values were imported into R version 4.3.1 ×64 bit (The R Project for Statistical Computing) and visualized using a heatmap and bar plot. Since each cytokine array chip can detect 2 independent serum samples, 2 chips were used to analyze the 4 serum samples from each group (corresponding to either serum from Vhl^f/f^ mice or from Vhl CKO mice).

### Data analysis

All data representative of three independent experiments are presented as the mean ± SEM. We used two-tailed *t-*tests to determine the significance of differences between two groups. We analyzed multiple groups by one- or two-way ANOVA with a Bonferroni post hoc correction in GraphPad Prism version 5. For all the statistical tests, we considered a *p* value < 0.05 to indicate statistical significance.

## Results

### *Vhl* ablation in osteoblasts causes increased bone density and HIF-1α activation

To further investigate the impact of the osteoblastic HIF-1α pathway on body metabolism, we crossed osteocalcin-Cre (Ocn-Cre) mice with *Vhl*^*flox/flox*^ mice to generate mice with constitutive HIF-1α pathway activation in osteoblasts, that is, *Vhl*^*flox/flox*^-Ocn-Cre^+/−^ mice (referred to as *Vhl* cKO mice). *Vhl*^*flox/flox*^-Ocn-Cre^−/−^ (referred to as *Vhl*^*flox/flox*^ mice) littermates were used as controls.

First, to determine the specificity of Ocn-Cre in targeting skeletal cells, we crossed Ocn-Cre transgenic mice with Rosa26;mT/mG reporter mice. These reporter mice switch from red fluorescent protein to green fluorescent protein (GFP) expression following Cre-mediated recombination. As expected, the bones of these mice showed GFP^+^ cells in the hypertrophic chondrocyte regions of the growth plate, throughout the metaphysis, and on and around the cortical (Cort) and trabecular (Trab) bone surfaces (Supplementary Fig. 1a). However, we did not observe any GFP^+^ cells in other tissues, such as the heart, liver, spleen, lung, kidney, brain, pancreas, thymus, stomach, duodenum, small intestine, colon, skeletal muscle, skin, testis, or ovary (Supplementary Fig. 1b). Additionally, the mRNA expression results further confirmed that bone tissues had the highest expression of Ocn compared to other tissues, as Ocn was barely detected in other tissues (Supplementary Fig. 1c). These results were consistent with the findings of a previous study by Dirckx et al.^28^, which evaluated the targeting of osteoprogenitors using the Osx (SP7)-Cre;GFP system in bone and soft tissues. Therefore, our findings confirm the skeletal specificity of Ocn-Cre.

Consistent with previous reports^20^, the *Vhl* cKO mice exhibited significantly greater bone mass. Microcomputed tomography (micro-CT) revealed an imbalance in the trabecular bone microstructure of the *Vhl* cKO mice (Supplementary Fig. 2a), as indicated by a substantial increase in bone density (BMD), bone volume/tissue volume (BV/TV), trabecular number (Tb.N), and trabecular thickness (Tb.Th) and a decrease in trabecular spacing (Tb.Sp) (Supplementary Fig. 2b–f). Hematoxylin and eosin (HE) staining revealed that almost all the bone marrow spaces in the long bones of the *Vhl* cKO mice were filled with new bone (Supplementary Fig. 2g), as confirmed by determination of the bone area/tissue area (Supplementary Fig. 2h). Further detection with Goldner staining revealed that most of the newly formed cancellous bone exhibited immature osteoid deposition in the mutant bone (Supplementary Fig. 2i). Deletion of *Vhl* blocks the oxygen-dependent inactivation of HIF, thus representing a model of constitutive HIF-1α signaling pathway activation^21,35,47^. Immunohistochemical (IHC) staining revealed that *Vhl* deletion increased HIF-1α expression in osteoblast lineage cells on and around the trabecular surfaces (Supplementary Fig. 2j), as confirmed by the average integrated density determined by IHC (Supplementary Fig. 2k).

### *Vhl* cKO mice are lean and exhibit hypoglycemia and increased glucose tolerance, with no changes in serum insulin levels or pancreatic β cells

The *Vhl* cKO mice displayed distinct alterations in whole-body homeostatic processes. The mutant mice had a lean appearance (Supplementary Fig. 3a), showing a marked reduction in the epididymal fat pad mass (Supplementary Fig. 3b). The *Vhl* cKO mice exhibited resistance to age-related weight gain and had significantly lower body weights than the control mice at 4 weeks of age (Supplementary Fig. 3c). These changes were accompanied by decreased blood glucose levels under random-feeding and fasting conditions (Supplementary Fig. 3d, e). The hypoglycemic phenotype was also characterized by increased glucose clearance from the blood circulation after an intraperitoneal glucose injection during a glucose tolerance test (GTT) (Supplementary Fig. 3f). Consistent with previous reports^28^, no alterations were observed in the serum insulin levels of the *Vhl* cKO mice compared to those of the controls (Supplementary Fig. 3g), despite the reduced blood glucose and increased glucose tolerance. Pancreatic β cells are the only insulin-producing cells that secrete insulin to control systemic glucose metabolism^48^. We found that, compared with those in the control mice, the number of islets and the expression of insulin in the *Vhl* cKO mice did not significantly change (Supplementary Fig. 3h–k), and the proliferation and apoptosis of pancreatic β cells also did not significantly change (Supplementary Fig. 3l–o). These results indicated that the deletion of *Vhl* in osteoblasts leads to hypoglycemia and increased glucose tolerance, with no changes in serum insulin levels or pancreatic β cells.

### *Vhl* deletion in osteoblasts partially protects mice from STZ-induced T1DM

**Fig. 1:**
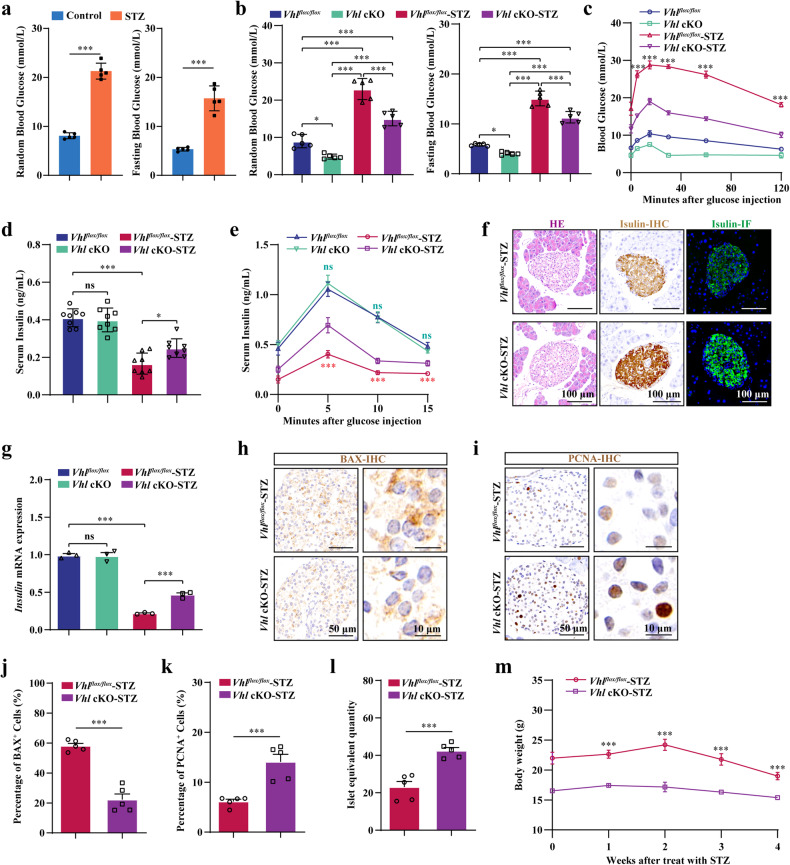
*Vhl* cKO partially protects mice from STZ-induced T1DM. **a** Random and fasting blood glucose levels of the 8-week-old control and STZ-treated mice (*n* = 5). **b** Random and fasting blood glucose levels of the 8-week-old *Vhl* cKO mice (*n* = 5) and their littermate controls (*n* = 5) after STZ injection. **c** GTTs of the 8-week-old *Vhl* cKO mice (*n* = 5) and their littermate controls (*n* = 5) after STZ injection. **d** Serum insulin levels of the 8-week-old *Vhl*^*flox/flox*^ mice (*n* = 5), *Vhl* cKO mice (*n* = 5), STZ-treated *Vhl*^*flox/flox*^ mice (*n* = 8) and STZ-treated *Vhl* cKO mice (*n* = 7). **e** The *Vhl* cKO mice exhibited greater glucose-stimulated insulin secretion (GSIS) than the control mice under STZ conditions. **f** HE staining and insulin immunostaining of pancreatic islets from the 8-week-old *Vhl* cKO mice and their littermate controls after STZ injection. **g** mRNA expression of insulin in the pancreatic islets of the 8-week-old *Vhl* cKO mice and their littermate controls after injection with STZ. **h**, **j** BAX IHC of pancreatic islets from the 8-week-old *Vhl* cKO mice and their littermate controls after STZ injection. **i**, **k** PCNA IHC of pancreatic islets from the 8-week-old *Vhl* cKO mice and their littermate controls after STZ injection (*n* = 5). **l** The islet equivalent (IEQ) of the Vhl cKO mice was greater than that of their control littermates after STZ treatment (*n* = 5). **m** Body weight changes in the *Vhl* cKO mice and their littermate controls after STZ injection. All data are presented as the mean ± SEM, and *p* values were analyzed by two-tailed *t-*tests in (**a**, **j**–**l**); one-way ANOVA in (**b**, **d**, **g**); and two-way ANOVA in (**c**, **e**, **m**). *p < 0.05, ***p < 0.001. All the data are representative of two to three independent experiments. The data were obtained from male mice.

Disorders in glucose metabolism commonly result in one of the most prevalent conditions related to high blood glucose, diabetes, which is mainly characterized by abnormally high glucose concentrations^49^. The hypoglycemic symptoms observed in *Vhl* knockout mice at basal levels prompted us to further investigate the ability of Vhl to regulate glucose metabolism in pathological states such as diabetes. Multiple low doses of intraperitoneal STZ were used to induce T1DM^50^. As shown in Fig. 1a, the blood glucose levels of the diabetic control mice progressively increased compared to those of the normal mice under both random-fed and fasted conditions, suggesting the successful induction of T1DM. Interestingly, the results also indicated that *Vhl* deletion in osteoblasts partially inhibited the rapid increase in serum glucose after STZ injection, which indicates increased glucose clearance during T1DM (Fig. 1b). In addition, the results of the GTT in the STZ-treated mice further confirmed the improved glucose clearance activity of the *Vhl* cKO mice (Fig. 1c).

T1DM occurs mainly due to a lack of adequate insulin, which results in increased serum glucose. We next compared the insulin content in the serum of the T1DM *Vhl* cKO mice and their T1DM littermate controls. The ELISA results showed a notable decrease in the serum insulin level of the diabetic control animals compared to that of the normal animals; however, *Vhl* deletion partially inhibited the STZ-induced decrease in serum insulin levels (Fig. 1d). Compared with the control mice, the *Vhl* cKO mice exhibited greater glucose-stimulated insulin secretion (GSIS) under STZ conditions (Fig. 1e). The expression of insulin in pancreatic β cells, as determined by IHC and immunofluorescence (IF) staining (Fig. 1f), as well as its mRNA expression (Fig. 1g), further confirmed that *Vhl* deletion partially suppressed the decrease in insulin induced by STZ. Multiple low doses of intraperitoneal STZ can induce insulin-dependent T1DM by causing the apoptosis of pancreatic β cells^51^. Fourteen days after the last STZ injection, more insulin-producing cells were found in the *Vhl* cKO mice than in the controls. IHC staining of BAX revealed that *Vhl* deficiency in osteoblasts partially decreased the STZ-induced apoptosis of β cells (Fig. 1h, j). In contrast, IHC staining of PCNA showed that pancreatic islet β cells in the *Vhl* cKO mice exhibited greater proliferation than those in the control mice (Fig. 1i, k). Consistent with the partial protection of the Vhl cKO mice against STZ-induced damage to pancreatic β cells, the islet equivalent (IEQ) analysis revealed a greater percentage of equivalent islets in the *Vhl* cKO mice than in their control littermates (Fig. 1l). Additionally, *Vhl* cKO partially inhibited the STZ-induced decrease in body weight 3 weeks after the last STZ injection (Fig. 1m).

To further confirm the protective effect of skeletal *Vhl* deletion on STZ-induced T1DM, we used a recently generated *Dmp-1*-Cre-driven *Vhl* cKO mouse model^35^. Late osteoblasts and osteocytes are specifically targeted in this mouse model, resulting in increased bone mass and activation of HIF-1α in these cells. Consistent with the phenotypes observed in the *Ocn*-Cre;*Vhl*^flox/flox^ mice, the *Dmp-1*-Cre;*Vhl*^flox/flox^ mice also had a lean appearance, hypoglycemia, and improved glucose tolerance, without any changes in serum insulin levels or pancreatic β cells. Furthermore, deletion of *Vhl* in late osteoblasts and osteocytes by *Dmp-1*-Cre partially improved glucose metabolism in STZ-induced diabetes. This deletion increased the ability to clear blood glucose and partially reduced blood glucose levels while also partially protecting pancreatic β cells from STZ-induced apoptosis, promoting their proliferation and increasing insulin secretion (Supplementary Fig. 4).

Taken together, these results demonstrate the partial protective effect of *Vhl* deletion against STZ-induced T1DM.

### *Vhl* deletion in osteoblasts promotes the expression of RegIIIγ and its circulation in the bloodstream

**Fig. 2:**
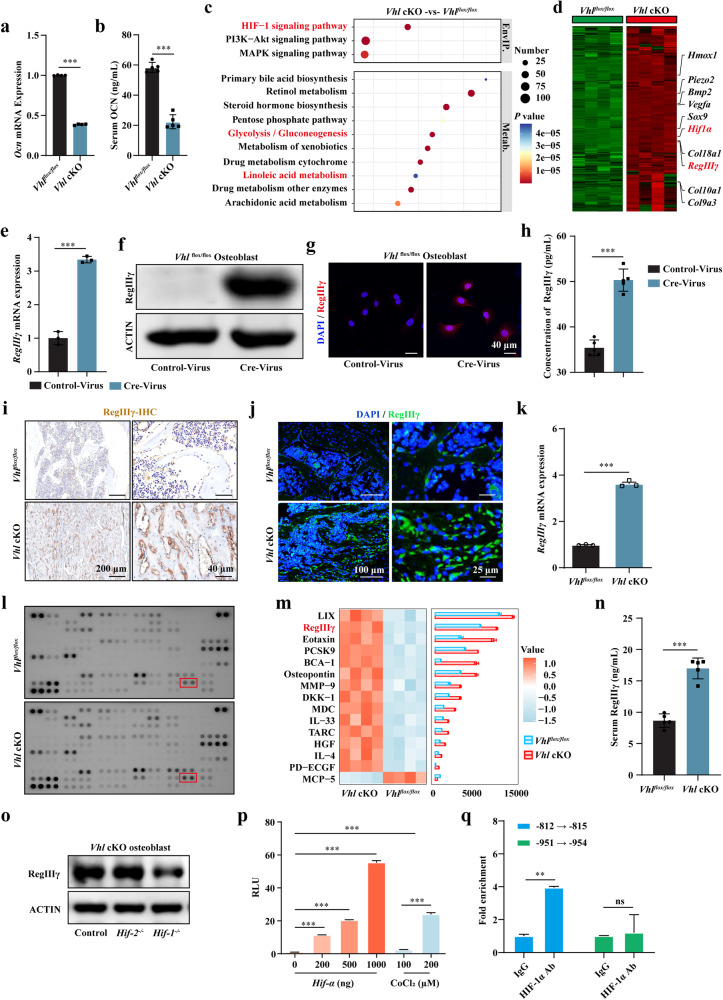
*Vhl* deletion in osteoblasts promotes the expression of RegIIIγ. **a** *Ocn* mRNA expression in the bone tissue of the *Vhl* cKO mice and their littermate controls (*n* = 4). **b** Levels of OCN in the serum of the *Vhl* cKO mice and their littermate controls (*n* = 5). **c** KEGG enrichment analysis of differentially expressed mRNAs between the bone tissue of the *Vhl* cKO mice and their littermate controls (*n* = 4). **d** Heatmap of mRNA expression in the bone tissue of the *Vhl* cKO mice and their littermate controls (*n* = 4). **e** RT‒qPCR analysis of *RegIIIγ* mRNA expression in vitro in the *Vhl*^*flox/flox*^ osteoblasts infected with Cre or control adenovirus (*n* = 3). **f** WB analysis of RegIIIγ expression in vitro in the *Vhlf*^*lox/flox*^ osteoblasts infected with Cre and control adenoviruses. **g** IF staining showing RegIIIγ expression in vitro in the *Vhl*^*flox/flox*^ osteoblasts infected with Cre and control adenoviruses. **h** ELISA analysis of the in vitro level of secreted RegIIIγ in the *Vhl*^*flox/flox*^ osteoblasts infected with Cre and control adenoviruses (*n* = 5). **i**, **j** Representative images of RegIIIγ IHC and IF staining of bone tissue from the *Vhl* cKO mice and their littermate controls. **k** Increased RegIIIγ mRNA expression was also found in the bone tissues of the *Vhl* cKO mice. **l** Cytokine microarray analysis of differentially expressed proteins in the serum of the *Vhl* cKO mice and their littermate controls (*n* = 4). **m** Differential enrichment analysis of serum cytokines between the *Vhl* cKO mice and their littermate controls (*n* = 4). **n** Serum RegIIIγ levels in the *Vhl* cKO mice and their littermate controls (*n* = 5). **o** Expression of RegIIIγ after *Hif-1α* or *Hif-2α* knockout in the *Vhl* knockout osteoblasts. **p** Dual-luciferase reporter assays to identify the Hif-1α-induced increase in RegIIIγ promoter activity. **q** ChIP‒qPCR analysis of the binding site of HIF-1α to the *RegIIIγ* promoter. All data are presented as the mean ± SEM, and *p* values were analyzed by two-tailed *t-*tests for (**a**, **b**, **e**, **h**, **k**, **n**, **q**) and one-way ANOVA for (**p**). ns, with no significant difference, **p < 0.01, ***p < 0.001. All the data are representative of two to three independent experiments. The data were obtained from male mice.

As an endocrine organ, bone regulates various metabolic processes, such as glucose metabolism, by secreting several cytokines^6,52^. In its undercarboxylated form, OCN is released by osteoblasts as a hormone into circulation. This molecule has been shown to promote pancreatic β cell proliferation, insulin secretion, and insulin sensitivity in peripheral tissues^6^. However, consistent with previous reports^28^, the *Ocn* mRNA expression in bone tissue and OCN protein expression in serum were significantly decreased in the *Vhl* cKO mice (Fig. 2a, b), which suggests that the protective effect of *Vhl* deletion against T1DM cannot be attributed to OCN. Another osteoblast-derived factor that promotes pancreatic β cell proliferation is lipocalin 2 (LCN2)^4,52^. However, the expression of *Lcn2* mRNA in bone tissue and the LCN2 protein in serum were also significantly decreased in the *Vhl* cKO mice (Supplementary Fig. 5a, b). Therefore, the protective effect of *Vhl* deletion against T1DM cannot be attributed to LCN2 either.

To further investigate which factors derived from osteoblasts are involved in the protective effect of *Vhl* deletion against diabetes, we performed whole-transcriptome sequencing of RNA from the bone tissues of the *Vhl* cKO mice and their littermate controls. Among the significantly and differentially expressed transcripts, 2100 genes were downregulated (<0.66-fold, *p* < 0.05) and 2674 genes were upregulated (>1.5-fold, *p* < 0.05) in the *Vhl* cKO mice compared with their littermate controls (Supplementary Fig. 6a). Kyoto Encyclopedia of Genes and Genomes (KEGG) and Gene Ontology (GO) enrichment analysis revealed that in addition to the HIF-1α pathway and bone trabecular formation, metabolic signaling pathways associated with glycol metabolism, lipid metabolism, etc., were strongly correlated with differentially expressed mRNAs between the *Vhl* cKO mice and their littermate controls (Fig. 2c and Supplementary Fig. 6d). Upon further analysis, we discovered that, in addition to *Hif-1α* and its downstream genes vascular endothelial growth factor (*Vegf*) and heme oxygenase 1 (*Hmox-1*), the increased expression of *RegIIIγ* in the *Vhl* cKO mice was particularly noteworthy (Fig. 2d and Supplementary Fig. 6b), as RegIIIγ has been proven to significantly contribute to pancreatic β cell regeneration and preserve pancreatic β cells from damage^53^. To further confirm whether *Vhl* knockdown in osteoblasts promotes RegIIIγ expression, we infected *Vhl*^*flox/flox*^ osteoblasts with Cre adenovirus. Real-time PCR (RT‒PCR), western blotting (WB) and IF showed a significant increase in RegIIIγ expression in the *Vhl*^*flox/flox*^ osteoblasts infected with Cre adenovirus (Fig. 2e–g). In addition, ELISAs consistently showed a substantial increase in the secreted RegIIIγ protein level in the *Vhl*^*flox/flox*^ osteoblasts infected with Cre adenovirus (Fig. 2h and Supplementary Fig. 6c). Consistent with the in vitro study, IHC and IF staining further confirmed the increased RegIIIγ expression in osteoblast lineage cells on and around the trabecular surfaces of the *Vhl* cKO mice (Fig. 2i, j). Additionally, increased RegIIIγ mRNA expression was detected in the bone tissues of the *Vhl* cKO mice (Fig. 2k). A proteome cytokine array further identified differential cytokine levels, revealing increased RegIIIγ circulation in the serum of the *Vhl* cKO mice (Fig. 2l, m and Supplementary Fig. 7). The increase in the serum RegIIIγ concentration was further confirmed using ELISAs (Fig. 2n). Additionally, increased RegIIIγ expression was observed in both the blood and bone tissue of the *Dmp-1*-Cre;*Vhl*^f/f^ mice (Supplementary Fig. 8). These results indicated that the upregulated RegIIIγ in *Vhl*-deficient osteoblasts can be released into the blood circulation.

*Vhl* deletion suppresses the degradation of HIF-1α and HIF-2α^24^. We observed that knocking out *Hif-1*α, rather than *Hif-2*α, significantly inhibited the upregulation of RegIIIγ expression induced by *Vhl* deletion in osteoblasts (Fig. 2o). As HIF-1α accumulates and translocates into the nucleus, this molecule can form a dimer with the HIF-1β subunit via its bHLH-PAS domain and bind to the promoter region of target genes, thereby increasing their expression^52^. Based on this mechanism, we speculated that HIF-1α binds to the promoter of *RegIIIγ* and stimulates its expression. To verify this hypothesis, we conducted a dual-luciferase reporter gene assay to measure the promoter activity of *RegIIIγ* in the presence of HIF-1α activation. The results showed that in parallel with HIF-1α and *RegIIIγ* upregulation, *Hif-1*α transfection increased *RegIIIγ* promoter activity in a dose-dependent manner (Fig. 2p). Furthermore, we investigated the location of the hypoxia-reactive element (HRE) in the *RegIIIγ* promoter region. By analyzing the promoter sequence of *RegIIIγ* with the JASPAR core database, we discovered two putative binding sites for *Hif-1α* at −951 → −954 bp (sense strand) and −812 → −815 bp (antisense strand) bp in the promoter region of *RegIIIγ*. To further investigate which putative binding site is crucial for HIF-1α-mediated transcription of *RegIIIγ*, we performed chromatin immunoprecipitation (ChIP) of the HIF-1α protein, and RT‒qPCR was performed to identify the enrichment of HIF-1α at the putative binding sites. The results revealed that the binding site of the HIF-1α transcription factor in the promoter of *RegIIIγ* was −812 → −815 rather than −951 → −954 (Fig. 2q). These data demonstrated that the deletion of *Vhl* in osteoblasts increases the expression of *RegIIIγ* in bone tissue and blood circulation.

### Osteoblastic-derived RegIIIγ assists mice in resisting STZ-induced T1DM

**Fig. 3:**
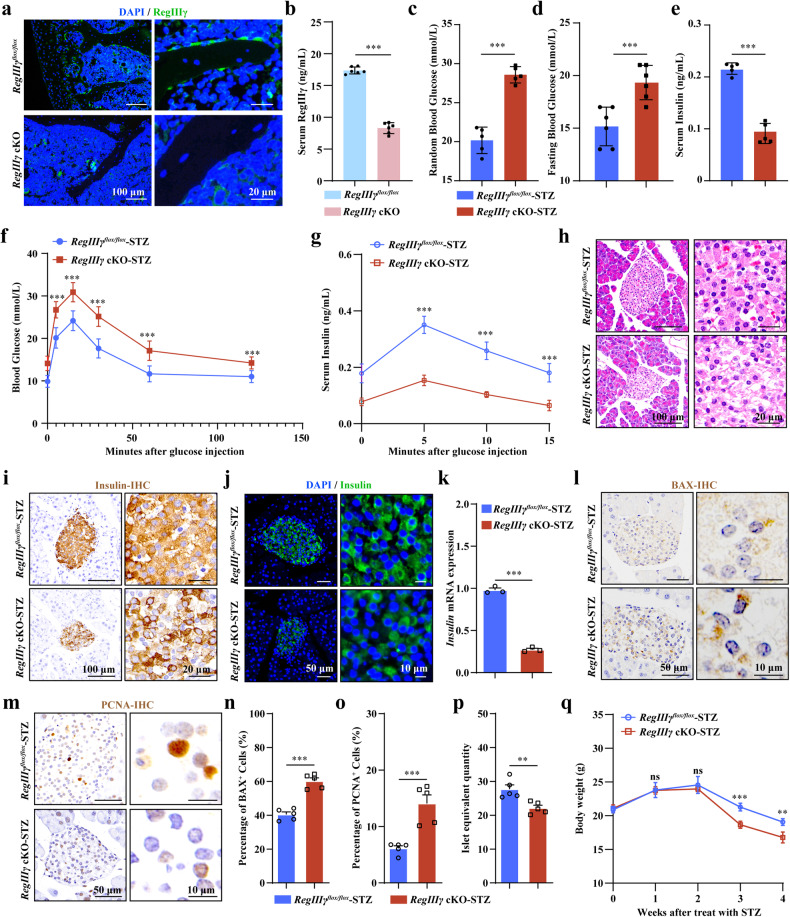
Osteoblastic-derived RegIIIγ assists mice in resisting STZ-induced T1DM. **a** Representative images of IF staining of RegIIIγ in bone tissue from the *RegIIIγ* cKO mice and their littermates. **b** Serum RegIIIγ levels in the *RegIIIγ* cKO mice and their littermate controls (*n* = 6). **c** Random blood glucose levels in the *RegIIIγ* cKO mice and their littermate controls after STZ induction (*n* = 5). **d** Fasting blood glucose levels in the *RegIIIγ* cKO mice and their littermate controls after STZ induction (*n* = 6). **e** Serum insulin levels in the *RegIIIγ* cKO mice and their littermate controls after STZ induction (*n* = 5). **f** GTTs of the *RegIIIγ* cKO mice and their littermate controls subjected to STZ induction (*n* = 6). **g** Compared with the control mice, the *RegIIIγ* cKO mice exhibited a decreased ability to increase GSIS under STZ conditions. **h** Representative images of HE-stained pancreatic islets from the *RegIIIγ* cKO mice and their littermate controls subjected to STZ induction. **i**, **j** Representative images of insulin IHC and IF staining of the *RegIIIγ* cKO mice and their littermate controls subjected to STZ induction. **k** Insulin mRNA expression in the pancreatic islets of the *RegIIIγ* cKO mice and their littermate controls after STZ induction (*n* = 3). **l**, **n** Pancreatic islet BAX IHC staining of the *RegIIIγ* cKO mice and their littermate controls after STZ induction. **m**, **o** PCNA IHC staining of pancreatic islets from the *RegIIIγ* cKO mice and their littermate controls after STZ induction. **p** The islet equivalent (IEQ) of the RegIIIγ cKO mice was lower than that of their control littermates after STZ treatment (*n* = 5). **q** Body weight changes in the *RegIIIγ* cKO mice and their littermate controls after STZ injection. All data are presented as the mean ± SEM, and *p* values were analyzed by two-tailed *t-*tests in (**b**–**e**, **k**, **n**–**p**) and two-way ANOVA in (**f**, **g**, **q**). **p < 0.01, ***p < 0.001. All the data are representative of two to three independent experiments. The data were obtained from male mice.

RegIIIγ is a protein primarily produced by the pancreas that plays a crucial role in the regeneration and preservation of pancreatic β cells, increases insulin production, and normalizes blood glucose control^53^. Having observed upregulated RegIIIγ expression in *Vhl*^*−/−*^ osteoblasts and increased RegIIIγ in the serum of *Vhl* cKO mice, we next investigated whether osteoblast-derived RegIIIγ has a protective role against T1DM. To generate mice with conditional deletion of *RegIIIγ* in osteoblasts, we bred Ocn-Cre mice with *RegIIIγ* floxed mice to generate *RegIIIγ*^*flox/flox*^-OCN-Cre^+/−^ mice (referred to as *RegIIIγ* cKO mice). For the control group, *RegIIIγ*^*flox/flox*^-Ocn-Cre^−/−^ littermates were used (referred to as *RegIIIγ*^*flox/flox*^ mice). IF staining of RegIIIγ in bone tissue confirmed the successful knockout of *RegIIIγ* in osteoblasts (Fig. 3a). *RegIIIγ* cKO mice were viable and born at the expected Mendelian ratio. The body size and weight of the *RegIIIγ* cKO mice were comparable to those of their littermate controls (Supplementary Fig. 9a).

Along with the knockout of *RegIIIγ* in osteoblasts, the RegIIIγ content in the blood also significantly decreased (Fig. 3b). Increased circulating glucose levels, both in random-fed and fasted conditions, were found in the serum of the *RegIIIγ* cKO mice with T1DM (Fig. 3c, d). Furthermore, the hyperglycemic phenotype observed in the *RegIIIγ* cKO mice with T1DM was characterized by impaired glucose clearance from the bloodstream, as indicated by the results of the GTT (Fig. 3f). In addition, compared with the control mice, the *RegIIIγ* cKO mice exhibited a decreased ability to increase GSIS under STZ conditions (Fig. 3g). Consistent with the decrease in glucose clearance, a decrease in the serum insulin concentration was found in the diabetic *RegIIIγ* cKO mice compared to the control mice (Fig. 3e). Further pathological examination revealed a significant increase in apoptosis and a notable decrease in the proliferation of pancreatic β cells (Fig. 3l–o), which led to a substantial decrease in the number and volume of pancreatic islets (Fig. 3h, p), along with a reduction in insulin expression in the β cells of the *RegIIIγ* cKO mice with T1DM (Fig. 3i–k). In addition, greater weight loss was observed in the *RegIIIγ* cKO mice than in the control littermates 2 weeks after the final STZ treatment (Fig. 3q). In contrast to the observations in individuals with T1DM, blood glucose levels, glucose tolerance, circulation insulin levels, and pancreatic β cell mass were similar between the *RegIIIγ* cKO mice and their littermate controls under normal physical conditions (Supplementary Fig. 9b–m).

We further validated the protective role of RegIIIγ in maintaining insulin release and glucose clearance in response to STZ. We examined insulin secretion stimulated by glucose in isolated islets in response to treatment with the RegIIIγ protein. As shown in Supplementary Fig. 10, STZ strongly impaired the insulin mRNA expression in isolated islets (Supplementary Fig. 10a). However, this damage was alleviated in a manner dependent on the concentration of the RegIIIγ protein (Supplementary Fig. 10a). Under these conditions, regardless of whether a low concentration (2.8 mM) or a high concentration (16.7 mM) of glucose was used, the stimulated release of insulin was significantly greater in the group treated with RegIIIγ plus STZ than in the group treated with STZ alone (Supplementary Fig. 10b). This finding further confirmed the role of RegIIIγ in promoting insulin release.

Taken together, these data indicated that osteoblast-derived RegIIIγ plays a protective role against T1DM.

### *Vhl* cKO alleviates T1DM partially via RegIIIγ

**Fig. 4:**
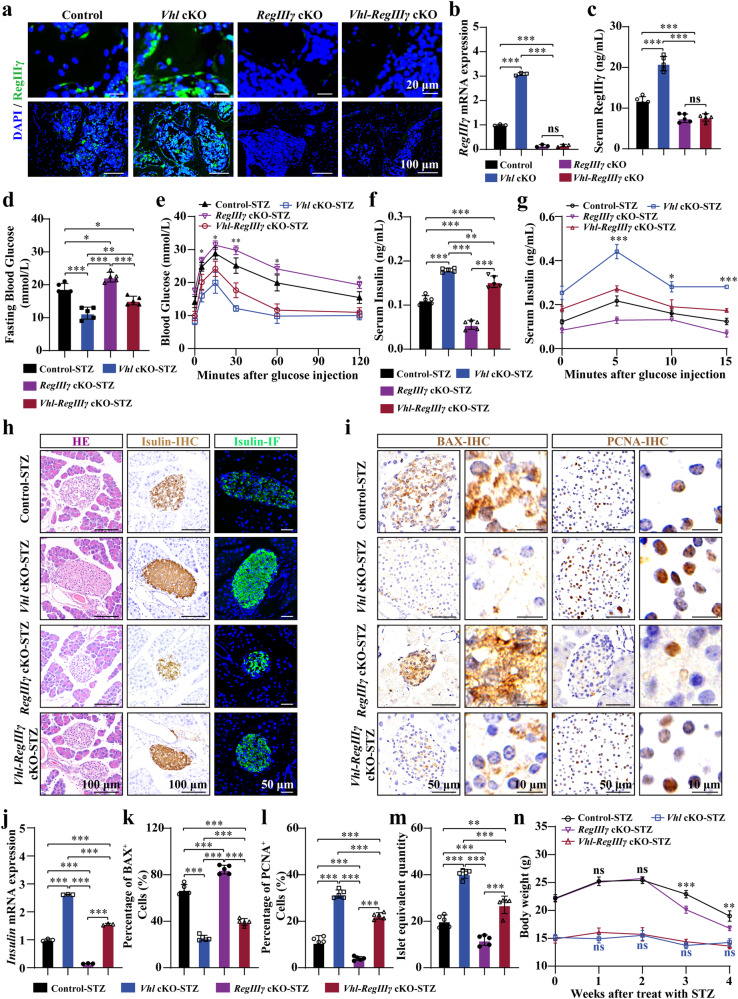
*Vhl* cKO alleviates T1DM partially via RegIIIγ. **a**, **b** RegIIIγ IF staining and RegIIIγ mRNA expression were analyzed in the control, *Vhl* cKO, *RegIIIγ* cKO, and *Vhl-RegIIIγ* cKO mice. **c** Serum RegIIIγ levels were measured in the control, *Vhl* cKO, *RegIIIγ* cKO, and *Vhl-RegIIIγ* cKO mice. **d** Fasting blood glucose levels were determined in the control, *Vhl* cKO, *RegIIIγ* cKO, and *Vhl-RegIIIγ* cKO mice subjected to STZ induction (*n* = 5). **e** GTTs were performed on the control, *Vhl* cKO, *RegIIIγ* cKO, and *Vhl-RegIIIγ* cKO mice subjected to STZ induction (*n* = 5). **f** Serum insulin levels were measured in the control, *Vhl* cKO, *RegIIIγ* cKO, and *Vhl-RegIIIγ* cKO mice after STZ induction (*n* = 5). **g** GSIS was assessed in the control, *Vhl* cKO, *RegIIIγ* cKO, and *Vhl-RegIIIγ* cKO mice after STZ induction (*n* = 5). **h** Representative images of pancreatic islets from the control, *Vhl* cKO, *RegIIIγ* cKO, and *Vhl-RegIIIγ* cKO mice subjected to STZ induction were subjected to HE staining and insulin immunostaining. **i**, **k**, **l** Islets of the control, *Vhl* cKO, *RegIIIγ* cKO, and *Vhl-RegIIIγ* cKO mice subjected to STZ induction were subjected to immunohistochemical (IHC) staining for BAX and PCNA. **j** Insulin mRNA expression was analyzed in the pancreatic islets of the control, *Vhl* cKO, *RegIIIγ* cKO, and *Vhl-RegIIIγ* cKO mice subjected to STZ induction (*n* = 3). **m** The islet equivalent quantity (IEQ) was calculated for the control, *Vhl* cKO, *RegIIIγ* cKO, and *Vhl-RegIIIγ* cKO mice subjected to STZ induction (*n* = 5). **n** Changes in body weight were measured in the control, *Vhl* cKO, *RegIIIγ* cKO, and *Vhl-RegIIIγ* cKO mice after STZ induction (*n* = 5). All data are presented as the mean ± SEM, and *p* values were analyzed by one-way ANOVA for (**b**–**d**, **f**, **j**–**m**) and two-way ANOVA for (**e**, **g**, **n**). *p < 0.05, **p < 0.01, ***p < 0.001. All the data are representative of two to three independent experiments. The data were obtained from male mice.

Having demonstrated the protective role of osteoblast-derived RegIIIγ against STZ-induced T1DM, we next investigated whether osteoblast-derived RegIIIγ contributed to the alleviated symptoms of T1DM observed in *Vhl* cKO mice. Mice with conditional deletion of osteoblastic *Vhl* and *RegIIIγ* were generated by crossing *Vhlf*^*lox/flox*^-Ocn-Cre^+/−^ mice (*Vhl* cKO mice) with *RegIIIγ*^*flox/flox*^-Ocn-Cre^+/−^ mice (*RegIIIγ* cKO mice) to generate *Vhlf*^*lox/flox*^-*RegIIIγ*^*flox/flox*^-Ocn-Cre^+/−^ mice (referred to as *Vhl-RegIIIγ* cKO mice). For the control group, *Vhlf*^*lox/flox*^-*RegIIIγ*^*flox/flox*^-Ocn-Cre^−/−^ littermates were used (referred to as *Vhl*-*RegIIIγ*^*flox/flox*^ mice). The knockout efficiency was confirmed through RegIIIγ immunostaining and analysis of RegIIIγ mRNA expression in bone tissues (Fig. 4a, b). *Vhl-RegIIIγ* cKO mice were viable and born at the expected Mendelian ratio, and their body size and weight were comparable to those of *Vhl* cKO mice.

Deletion of *RegIIIγ* in osteoblasts significantly hindered the increase in serum RegIIIγ observed in the *Vhl* cKO mice (Fig. 4c). The quantification of blood glucose revealed that the deletion of osteoblastic *RegIIIγ* partially inhibited the decrease in blood glucose and impaired glucose tolerance in the *Vhl* cKO mice with T1DM (Fig. 4d, e). In addition, *RegIIIγ* cKO partially impaired GSIS in the *Vhl* cKO mice (Fig. 4g). Insulin immunostaining and insulin mRNA expression, as well as the calculation of the IEQ, revealed that the protective effect of osteoblast Vhl knockout on pancreatic β cells was partially reduced in the *Vhl-RegIIIγ* cKO mice after STZ induction (Fig. 4h, j, m). Compared with the *Vhl* cKO mice, the diabetic Vhl-RegIIIγ cKO mice exhibited significantly lower serum insulin levels (Fig. 4f). IHC staining of BAX further revealed that the protective effect of osteoblastic *Vhl* deletion in preventing STZ-induced apoptosis of β cells was partially diminished by *RegIIIγ* deletion in osteoblasts (Fig. 4i, k). Additionally, the increased proliferation of pancreatic β cells observed in the *Vhl* cKO mice was partially inhibited by osteoblastic *RegIIIγ* deletion (Fig. 4i, l). However, no difference in body weight changes was observed between the *Vhl* cKO and *Vhl-RegIIIγ* cKO mice (Fig. 4n).

The blockade of RegIIIγ by a RegIIIγ-neutralizing antibody also partially impaired the protective effect of *Vhl cKO* on STZ-induced T1DM. This phenomenon was evident from the increase in blood glucose levels, decreased glucose tolerance, decreased serum insulin levels, decreased GSIS, decreased IEQ, and the observed increase in apoptosis and decrease in proliferation of β cells in the *Vhl* CKO mice (Supplementary Fig. 11).

Taken together, these data demonstrated that RegIIIγ partially contributed to the alleviated symptoms of T1DM that were observed in *Vhl* cKO mice.

### SF-DFO activates the osteoblastic HIF-1α-RegIIIγ pathway and partially alleviates the symptoms of T1DM in adult mice

**Fig. 5:**
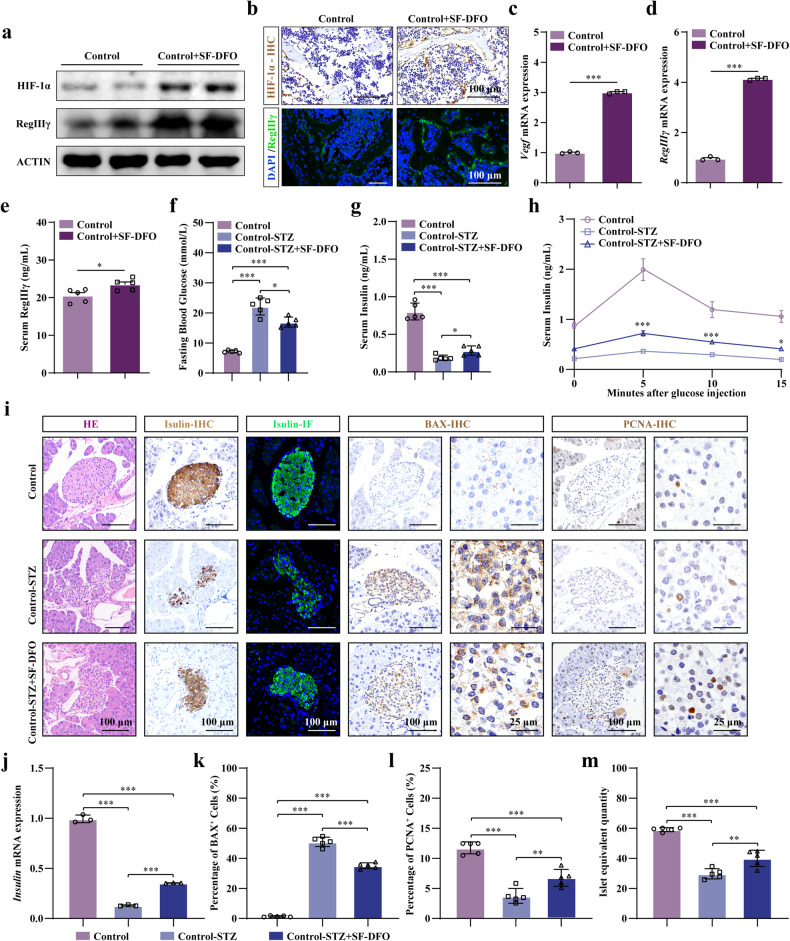
SF-DFO activates the osteoblastic HIF-1α-RegIIIγ pathway and partially alleviates the symptoms of T1DM in adult mice. **a** WB analysis of HIF-1α and RegIIIγ protein expression in the bone tissues of the mice treated with or without SF-DFO. **b** HIF-1α and RegIIIγ immunostaining in bone tissue. **c**, **d** VEGF and RegIIIγ mRNA expression in the bone tissues of the mice treated with or without SF-DFO (*n* = 3). **e** Serum RegIIIγ levels in the mice treated with or without SF-DFO (*n* = 5). **f** Fasting blood glucose in the mice treated with STZ/SF-DFO (*n* = 5). **g** Serum insulin levels in the mice treated with STZ/SF-DFO (*n* = 5). **h** GSIS of the mice treated with STZ/SF-DFO (*n* = 5). **i**, **k**, **l** Islet HE staining, insulin immunostaining, and insulin, BAX, and PCNA IHC staining images of the mice treated with STZ/SF-DFO (*n* = 5). **j** Insulin mRNA expression in the islets of the mice treated with STZ/SF-DFO (*n* = 3). **m** Calculation of islet equivalent quantity in the mice treated with STZ/SF-DFO (*n* = 5). All data are presented as the mean ± SEM, and *p* values were analyzed by two-tailed *t-*tests in (**c**–**e**); one-way ANOVA in (**f**, **g**, **j**–**m**); and two-way ANOVA in (**h**). *p < 0.05, **p < 0.01, ***p < 0.001. All the data are representative of two to three independent experiments. The data were obtained from male mice.

After observing the protective effect of activation of the osteoblastic VHL/HIF-1α-RegIIIγ pathway against STZ-induced T1DM in transgenic mice, we investigated its regulatory role in glucose metabolism in T1DM by activating the osteoblastic HIF-1α-RegIIIγ pathway using hypoxia-mimicking agents (HMAs). HMAs, such as deferoxamine (DFO), activate the HIF-1α pathway^54,55^. To specifically activate the HIF-1α pathway in bone tissue, we used iminodiacetic acid (IDA, NH(CH2COOH)2), a calcium chelating agent, as the lead compound to develop bone-seeking SF-DFO and found that SF-DFO can activate the HIF-1α pathway specifically in osteoblasts, promoting their osteogenic differentiation to prevent estrogen-induced bone loss and facilitate bone regeneration but without the inflammatory response observed in the liver and spleen^33^. Therefore, SF-DFO was used to stimulate the HIF-1α pathway in the present study in young 8-week-old mice. The WB results revealed that SF-DFO significantly increased the expression of HIF-1α and its downstream *RegIIIγ* (Fig. 5a). Immunostaining revealed elevated HIF-1α and RegIIIγ expression in osteoblast lineage cells located on and around the trabecular surfaces (Fig. 5b). Furthermore, elevated mRNA expression of *Vegf* and *RegIIIγ* was observed in the bone tissues of the mice treated with SF-DFO (Fig. 5c, d). Additionally, a greater level of RegIIIγ was observed in the serum following the administration of SF-DFO (Fig. 5e).

Having demonstrated the role of SF-DFO in activating the osteoblastic HIF-1α-RegIIIγ pathway, we next investigated whether SF-DFO alleviated the symptoms of T1DM. The analysis of blood glucose levels showed that SF-DFO partially inhibited the rapid increase in blood glucose induced by STZ in fasted states (Fig. 5f). Furthermore, SF-DFO was shown to be a significant stimulant of glucose-stimulated insulin secretion (GSIS) in the mice treated with STZ (Fig. 5h). In addition, the quantification of insulin in serum, the IHC and IF staining of insulin in pancreatic β cells and the mRNA expression of insulin in the pancreatic islets further revealed that SF-DFO partially suppressed the STZ-induced decrease in insulin (Fig. 5g, i, j). IHC staining of BAX and PCNA, as well as calculation of the islet equivalent quantity, consistently revealed that SF-DFO application partially aided pancreatic β cells in preventing STZ-induced apoptosis and promoted β cell proliferation (Fig. 5i, k‒m). Collectively, these data indicated that SF-DFO can activate the osteoblastic HIF-1α-RegIIIγ pathway and partially alleviate the symptoms of STZ-induced T1DM in mice.

### SF-DFO restores the impaired osteoblastic HIF-1α-RegIIIγ pathway and partially relieves the symptoms of T1DM in elderly mice

**Fig. 6:**
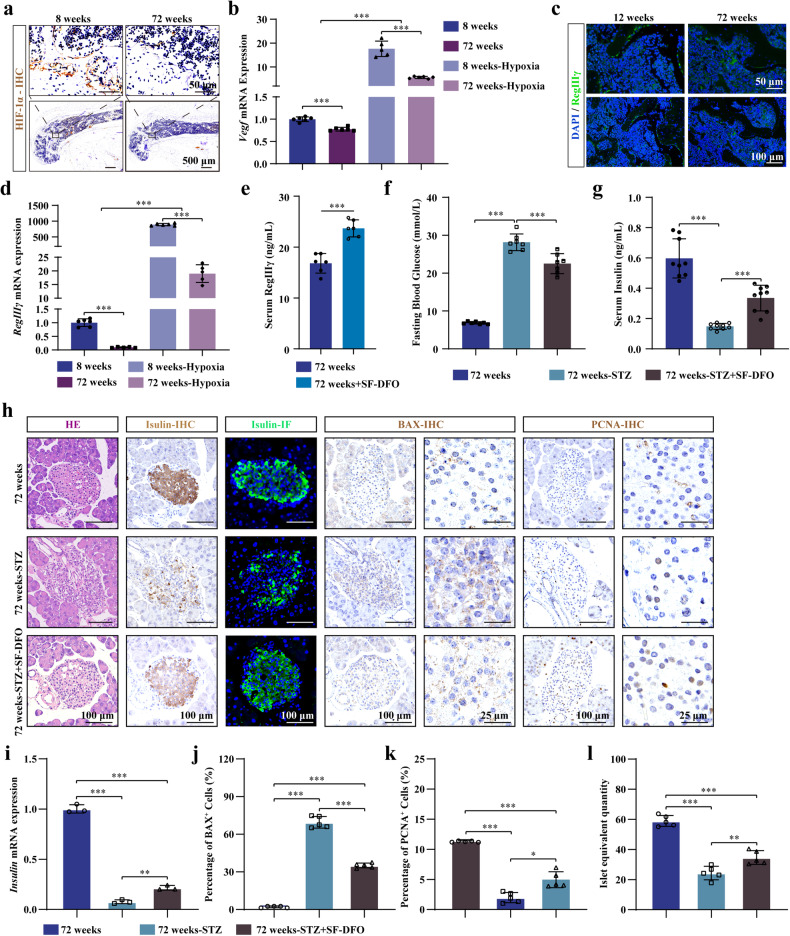
SF-DFO restores the impaired osteoblastic HIF-1α-RegIIIγ pathway and partially relieves the symptoms of T1DM in elderly mice. **a** HIF-1α IHC staining of femurs from 8-week-old and 72-week-old young and old mice. **b** VEGF mRNA expression in bone tissue from the young and old mice after the removal of bone marrow and incubation in an incubator with 1% oxygen for 24 h (*n* = 6). **c** Representative images of RegIIIγ IF staining in the young and old mouse femurs. **d** RegIIIγ mRNA expression in bone tissue from the young and old mice after the removal of bone marrow and incubation in an incubator with 1% oxygen for 24 h (*n* = 6). **e** Serum RegIIIγ levels in the old and SF-DFO-treated old mice (*n* = 6). **f** Fasting blood glucose in the old and SF-DFO-treated old mice after STZ-induced islet injury (*n* = 7). **g** Serum insulin levels in the old and SF-DFO-treated old mice after STZ-induced islet injury (*n* = 9). **h**, **j**, **k** Representative images of islet HE staining, insulin immunostaining, and BAX and PCNA IHC staining in the old and SF-DFO-treated old mice after STZ-induced islet injury. **i** Insulin mRNA expression in the islets of the SF-DFO-treated old mice after STZ-induced islet injury. **l** Calculation of the equivalent islet quantity in the SF-DFO-treated old mice after STZ-induced islet injury. All data are presented as the mean ± SEM, and *p* values were analyzed by two-tailed *t-*tests in (**e**) and one-way ANOVA in (**b**, **d**, **f**, **g**, **i**–**l**). *p < 0.05, **p < 0.01, ***p < 0.001. All the data are representative of two to three independent experiments. The data were obtained from male mice.

Aging is one factor that increases individuals’ susceptibility to diabetes^56^. As aging occurs, pancreatic function may decrease, leading to reduced or disrupted insulin secretion and ineffective use of glucose in the bloodstream, ultimately causing or exacerbating diabetes^57^. HIF-1α in elderly bone tissue is downregulated, and the responsiveness of elderly bone tissue to low oxygen is decreased^18^. Consistent with previous reports, our study also revealed significant decreases in HIF-1α expression in bone tissues from elderly mice (Fig. 6a) and decreased responsiveness to low oxygen in bone tissues from elderly 72-week-old mice, as demonstrated by decreased *Vegf* expression (Fig. 6b). In addition, our study revealed decreased *RegIIIγ* expression in the bone tissues of 72-week-old mice (Fig. 6c, d). Furthermore, the ability of aged bone tissue to express RegIIIγ in response to low-oxygen environments was significantly reduced (Fig. 6d). Although the ability of aged bone tissue to express RegIIIγ in response to low oxygen environments is reduced, *RegIIIγ* expression in low oxygen environments was far greater than that in normal oxygen environments (Fig. 6d). Therefore, we further investigated whether SF-DFO can alleviate diabetes symptoms in aged 72-week-old mice by restoring the HIF-1α-RegIIIγ pathway in osteoblasts. Serum RegIIIγ levels were significantly increased in aged mice after 1 month of treatment with SF-DFO (Fig. 6e). The results of quantitative tests for blood glucose concentration and insulin content in the bloodstream showed that SF-DFO significantly increased insulin levels and lowered glucose levels in the serum of elderly diabetic mice (Fig. 6f, g). Further histological staining showed that SF-DFO partially inhibited STZ-induced pancreatic β cell apoptosis and promoted pancreatic β cell proliferation and pancreatic β cell mass and insulin expression in diabetes (Fig. 6h–l). These results indicated that SF-DFO restores the impaired osteoblastic HIF-1α-RegIIIγ pathway and partially relieves the symptoms of T1DM in elderly mice.

## Discussion

Abnormal differentiation of osteoblasts, the primary cells regulating bone formation, leads to abnormal bone development and disrupts the endocrine functions of the skeleton^13,14,58^. The osteoblastic HIF-1α pathway plays a crucial regulatory role in bone development in oxygen-sensing cells^20,21^. Previous studies have shown that activation of the HIF-1α pathway increases glucose uptake by osteoblasts, which reduces blood glucose levels^28^. However, it is unclear whether activating the HIF-1α pathway in osteoblasts can help normalize glucose metabolism under diabetic conditions through its endocrine function. In addition to increasing bone mass and reducing blood glucose levels, this study revealed that activating the HIF-1α pathway by specifically knocking out *Vhl* in osteoblasts partially alleviated the symptoms of STZ-induced T1DM. Further screening of bone-derived factors revealed that RegIIIγ is an osteoblast-derived hypoxia-sensing factor that improves blood glucose clearance by protecting pancreatic β cells from STZ-induced apoptosis, promoting pancreatic β cell proliferation, and stimulating insulin secretion. In addition, we found that SF-DFO, a compound that mimics hypoxia and targets bone tissue, can partially alleviate symptoms of STZ-induced T1DM by activating the HIF-1α-RegIIIγ pathway in the skeleton. Thus, our data suggest that the osteoblastic HIF-1α-RegIIIγ pathway could be a potential target for treating T1DM.

Bone has been found to act as an endocrine organ that regulates various metabolic processes in the body, including glucose metabolism, immunity, lipid metabolism, and phosphorus metabolism^2^. Osteoblasts, which are responsible for bone formation, produce OCN, which stimulates insulin secretion, normalizes insulin sensitivity, and regulates male reproductive function^6–8^. Another protein, LCN2, suppresses appetite and regulates glucose metabolism and pancreatic islet function^4,52^. Fibroblast growth factor 23, which is secreted by osteoblasts, regulates phosphorus metabolism, while sclerostin regulates peripheral fat metabolism^9–12^. Recent studies have shown that interleukin 11, derived from osteoblasts and osteocytes, regulates fat metabolism^5^. Disruption of bone homeostasis can lead to various metabolic disorders, such as severe hematopoietic abnormalities^13^, abnormal immune regulation^14^, and disturbances in glucose metabolism^15^. Therefore, maintaining normal growth and differentiation of osteoblasts is crucial for bone homeostasis and metabolic processes.

Bone tissue cells grow and differentiate in a hypoxic microenvironment, and the HIF-1α pathway plays a critical role in regulating osteoblast differentiation and the secretion of bone-derived factors to regulate body metabolism^20,24,27^. Therefore, the coordinating role of the osteoblastic HIF-1α pathway in bone homeostasis and the endocrine function of the skeleton suggests that a thorough investigation of the regulatory role of the osteoblastic HIF-1α pathway in body metabolism is needed to increase our understanding of the endocrine function of bone tissue. The specific knockout of *Vhl*, a key molecule involved in the conversion of aerobic metabolism and glycolytic processes^59^, in osteoblasts resulted in persistent hypoglycemia and increased systemic glucose tolerance in mice due to increased osteoblast glucose uptake and glycolysis^28^. Another typical response to abnormal glucose metabolism is the high glucose symptoms that cause diabetes. However, it is unclear whether activating the HIF-1α pathway in osteoblasts can normalize glucose metabolism under diabetic conditions through its endocrine function. Consistent with a previous article^28^, the present study revealed that osteoblastic *Vhl* cKO mice exhibited a lean body size, reduced basal blood glucose levels, increased glucose tolerance, increased bone mass, slower differentiation from osteoblasts to osteocytes, and an abnormal increase in immature osteoids. By introducing the STZ-induced acute islet injury diabetic model, we further revealed that the activation of the hypoxic pathway by knocking out *Vhl* in osteoblasts partially alleviated the symptoms of STZ-induced diabetes, including reducing STZ-induced islet injury, increasing serum insulin levels, reducing blood glucose levels in the diabetic state and increasing glucose tolerance.

The protective effect of *Vhl* deletion in osteoblasts against STZ-induced islet injury prompted us to further investigate the bone-derived factors involved in this process. Previous studies have shown that osteoblast-derived OCN and LCN2 have similar regulatory effects on glucose metabolism and islets. Mice with osteoblasts overexpressing OCN experienced hypoglycemia and were protected against obesity and poor glucose tolerance due to increased pancreatic β cell proliferation, insulin secretion, and insulin sensitivity^6^. In contrast, mice with *Lcn2* gene-specific knockout in osteoblasts exhibited reduced glucose tolerance, decreased insulin sensitivity, and decreased insulin secretion^52^. In this study, we observed that osteoblasts with VHL knockout had lower serum levels of OCN and LCN2 than did their littermate controls, which suggests that factors other than OCN and LCN2 may play a role in regulating STZ-induced islet injury through the activation of the hypoxic pathway in osteoblasts. Through further screening, we identified RegIIIγ as an osteoblast-derived hypoxia-sensing factor that is partially responsible for improving blood glucose clearance by protecting pancreatic β cells from STZ-induced apoptosis, promoting pancreatic β cell proliferation and stimulating insulin secretion.

The regenerating islet-derived protein (REG), or pancreatic stone or pancreatitis-associated protein, belongs to the C-type lectin family^60^. RegIIIγ, the mouse homolog of human REG3A (also known as the human hepatocarcinoma-intestine pancreas or human pancreatitis-associated protein), has been found to attenuate the diabetic phenotype during STZ-induced islet damage and obesity-induced islet abnormalities by modulating pancreatic immunity and promoting pancreatic β cell regeneration^61,62^. Additionally, this molecule preserves the mitochondrial function of pancreatic β cells^63^. Recent research has also demonstrated that RegIIIγ functions as a nociceptor-enriched hormone that safeguards mice against endotoxin death by being secreted into the bloodstream by dorsal root ganglion cells^64^. Notably, our study revealed that osteoblasts secrete RegIIIγ, which is directly regulated by HIF-1α, a downstream target molecule. Alterations in RegIIIγ expression in osteoblasts can directly affect the circulation of RegIIIγ. As a result, this protein may function as a hormone-like molecule that protects against STZ-induced islet injury.

The critical role of the hypoxic pathway in bone modeling, remodeling, and regeneration has led to the development of medicinal HMAs. Deferoxamine (DFO), an FDA-approved iron chelator for treating iron toxicity or iron overload, activates the HIF-1α pathway and is widely used as an HMA under normoxic conditions^54,65–68^. However, DFO is water soluble, and several notable drug-related systemic toxicities, including cardiovascular, respiratory, gastrointestinal, dermal, and neurological toxicity, have been reported^69^. To target bone tissue, we constructed the bone-targeting hypoxic mimetic compound SF-DFO. SF-DFO was concentrated in bone tissue, activated HIF-1α, and showed better protected against bone loss in ovariectomized mice than DFO^33^, but no inflammatory response was observed in the liver or spleen^55^. Given the targeting and effectiveness of SF-DFO in activating the HIF-1α pathway in bone tissue cells, we examined whether SF-DFO regulates glucose metabolism in the diabetic state by activating the hypoxic pathway in osteoblasts. SF-DFO alleviated diabetic symptoms in young and old mice by activating the HIF-1α-RegIIIγ pathway in bone cells.

The incidence of osteoporosis and diabetes increases significantly with age, with osteoporosis being more prevalent than diabetes after age 70 years^70,71^. Based on our findings and previous reports that osteoblasts have significant regulatory effects on islets, we hypothesized that susceptibility to diabetes may increase as skeletal degeneration and osteoblast function decrease. Recent studies have suggested that skeletal degeneration may be a risk factor for developing diabetes mellitus, as treating osteoporosis with denosumab can substantially reduce diabetes^72^. Our findings and previous reports suggest that osteoblasts play an important role in regulating islets, and susceptibility to diabetes may increase as skeletal degeneration and osteoblast function decline with age. However, aging affects tissue cells throughout the body, and there is no single model of aging-related skeletal degeneration to confirm a direct relationship between skeletal degeneration and diabetes. Aged bone tissue exhibits reduced hypoxic sensitivity and reduced HIF-1α expression in osteoblasts, significantly decreasing bone mass and resulting in a phenotype similar to that of age-related osteoporosis^73^. *Vhl* deletion in osteoblasts results in a more active and youthful skeleton phenotype, increasing cell proliferation and promoting osteogenic activity^35^, indicating that activating the hypoxic pathway in osteoblasts may protect against diabetes by increasing osteoblastic activity. Indeed, the present study demonstrated that activating the HIF-1α pathway in osteoblasts by knocking out *Vhl* in osteoblasts or activating the bone tissue hypoxia pathway through SF-DFO promoted bone formation and partially reduced diabetes symptoms induced by STZ.

However, we would like to highlight some potential limitations of our study. First, although we investigated the endocrine function of the osteoblastic VHL/HIF-1α pathway on glucose metabolism and demonstrated that *Vhl* deletion in osteoblasts increases glucose clearance in diabetic mice by increasing RegIIIγ circulation in the blood, *Vhl* deletion in osteoblast lineage cells likewise elevated glucose uptake by the skeleton, effectively lowering blood glucose levels. It is still unclear whether this glucose-utilizing effect of *Vhl*-deficient osteoblasts contributes to glucose clearance in diabetic states. Second, the knockout of *Vhl* leads to the simultaneous activation of HIF-1α and HIF-2. HIF-1α, rather than HIF-2, plays a crucial role in bone formation^74^. Furthermore, our study revealed that HIF-1α, not HIF-2α, regulates RegIIIγ expression in osteoblasts. However, to further confirm that activation of the HIF-1α pathway partially occurs through RegIIIγ to prevent T1DM, it would be better to use mice with specific overexpression of HIF-1α in osteoblasts. Alternatively, determining whether osteoblast-specific *Hif-1*α knockout mice are more sensitive to T1DM due to decreased RegIIIγ expression in osteoblasts would also be helpful. Third, the findings of this study were obtained from male mice. Since there are well-documented and significant variations in bone mass and energy metabolism based on sex^75–77^, additional experiments were conducted on female mice to investigate the potential sex disparities in our findings. Supplementary Fig. 12 shows that female *Vhl* cKO mice also experienced a significant increase in bone mass (Supplementary Fig. 12a). This finding is indicated by higher values of BMD, BV/TV, Tb.N, and Tb.Th, as well as a decrease in Tb.Sp (Supplementary Fig. 12b). Moreover, the *Vhl* cKO female mice exhibited hypoglycemia and no changes in serum insulin levels or basal glucose clearance ability (Supplementary Fig. 12c–e). Additionally, the *Vhl* cKO female mice exhibited partial protection against STZ-induced T1DM. This protection included improved glucose clearance and GSIS in the presence of STZ, the preservation of pancreatic β cells from STZ-induced apoptosis, the stimulation of pancreatic β cell proliferation, increased insulin secretion, and reduced body weight loss (Supplementary Fig. 12c–l). Furthermore, we observed elevated expression of RegIIIγ in the femurs and increased levels of RegIIIγ in the serum of the *Vhl* cKO female mice (Supplementary Fig. 12m, n). These results indicate that *Vhl* knockout promotes bone formation and partially improves glucose metabolism in both male and female mice with STZ-induced T1DM. However, further research is needed to determine whether osteoblastic-derived RegIIIγ plays a protective role against T1DM in the *Vhl*-deficient female mice despite the elevated expression of RegIIIγ in the femurs and increased levels of RegIIIγ in the serum of the *Vhl* cKO female mice.

**Fig. 7:**
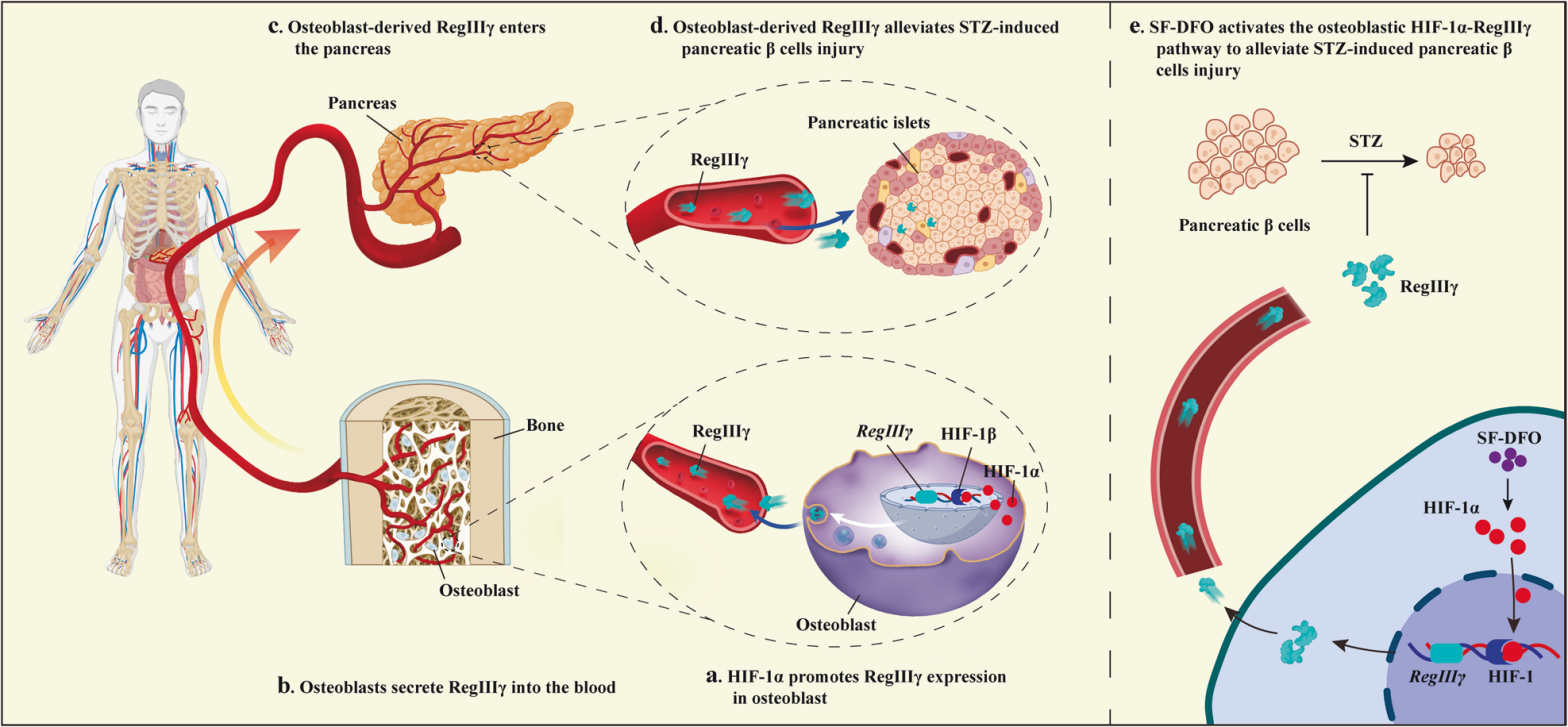
Schematic showing how activation of the osteoblastic HIF-1α pathway alleviates the symptoms of STZ-induced T1DM via RegIIIγ. **a** HIF-1α increases RegIIIγ gene expression by binding to the promoter region of *RegIIIγ*. **b** RegIIIγ secreted by osteoblasts enters the blood circulation. **c** Osteoblast-derived RegIIIγ enters the pancreas through the blood circulation. **d** Osteoblast-derived RegIIIγ decreases STZ-induced β cell injury. **e** SF-DFO activates the osteoblastic HIF-1α-RegIIIγ pathway to alleviate STZ-induced pancreatic β cell injury. This scheme was created with BioRender.com.

In summary, our study demonstrated that activating the HIF-1α pathway in osteoblasts can promote glucose metabolism under diabetic conditions via RegIIIγ and suggested that the osteoblastic HIF-1α-RegIIIγ pathway is a potential target for treating T1DM (Fig. 7).

### Supplementary information

Supplementary Materials

Supplementary Table 7

## Data Availability

The data supporting the findings of this study are available from the corresponding author upon reasonable request.
