# Supplementary material for: Activation of the osteoblastic HIF-1α pathway partially alleviates the symptoms of STZ-induced type 1 diabetes mellitus via RegIIIγ
Source: Exp Mol Med. 2024 Jul 1;56(7):1574–90. doi: 10.1038/s12276-024-01257-4 (PMC11297314; doi:10.1038/s12276-024-01257-4)
Supplement: Supplementary file 1 — Supplementary Materials [file 12276_2024_1257_MOESM1_ESM.pdf]

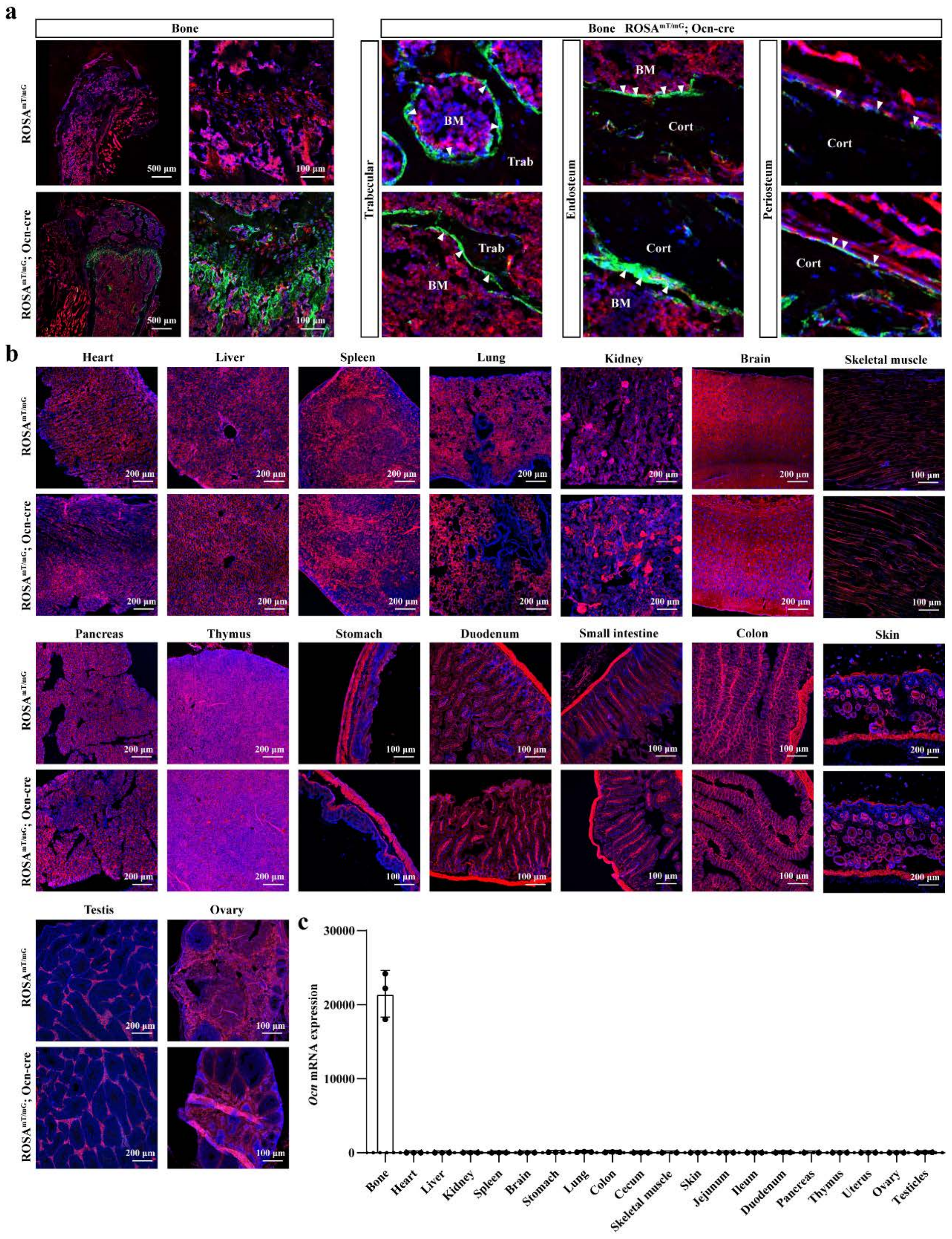

Supplementary Fig. 1. Reporter readout of *Ocn*-Cre activity in bones and soft tissues. (a) The bone of these mice showed GFP^{+} cells in the hypertrophic chondrocyte regions of the growth plate, throughout the metaphysis, and on and around the cortical (Cort) and trabecular (Trab) bone surface. **(b)** GFP^{+} cells in other tissues such as the heart, liver, spleen, lung, kidney, brain, pancreas, thymus, stomach, duodenum, small intestine, colon, skeletal muscle, skin, testis, and ovary. **(c)** the mRNA expression

results revealed that bone tissues have the highest expression of *Ocn* compared to other tissues, as *Ocn* is barely detected in other tissues. The data were obtained from male mice.

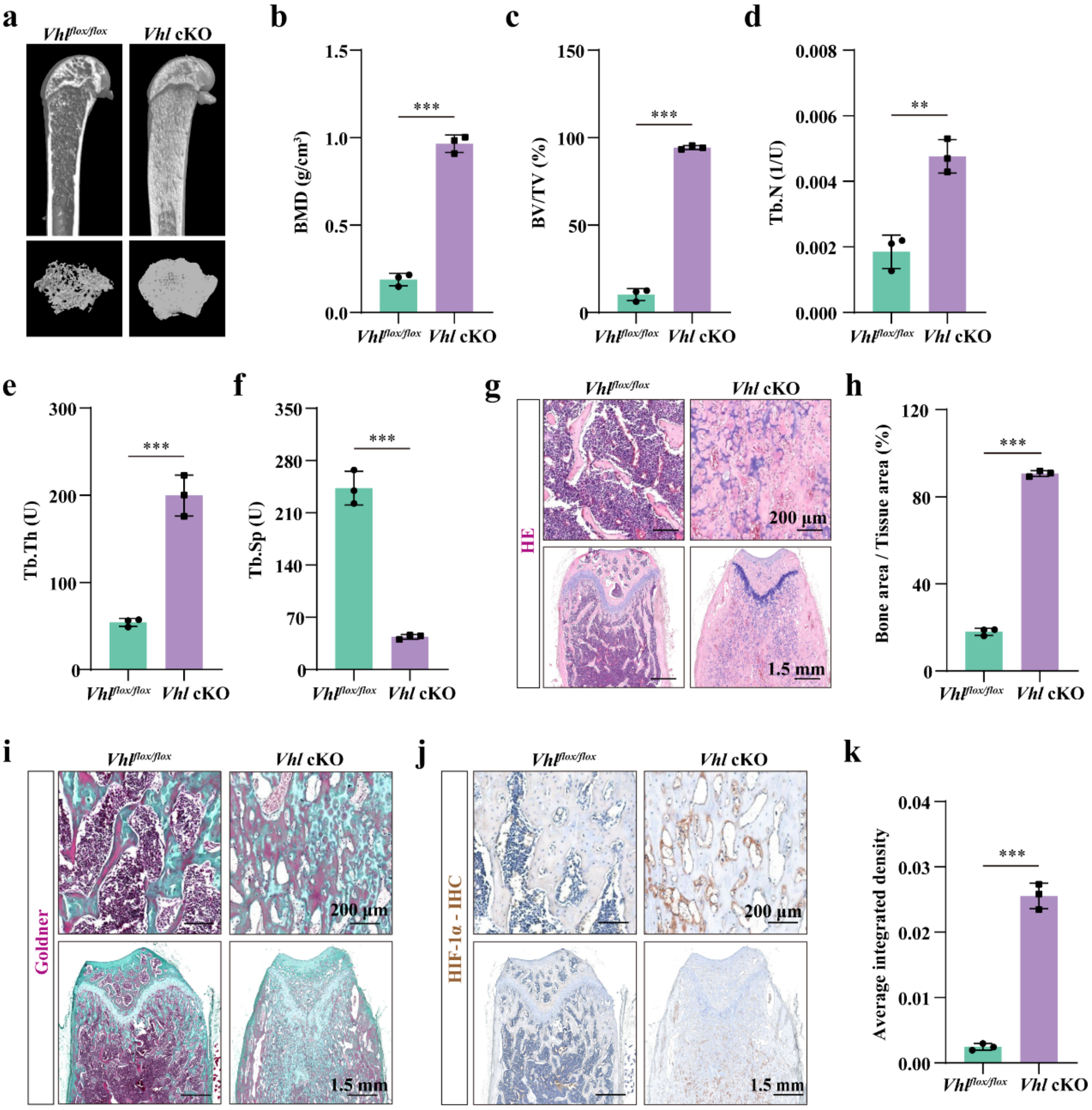

Supplementary Fig. 2. *Vhl* ablation in osteoblast accelerates the bone formation and activates the HIF-1α pathway. (a) Representative Micro-CT images of 8-week-old *Vhl* cKO mice and their littermate control femurs. **(b - f)** Bone trabecular analysis parameters, including BMD, BV/TV, Tb.N, Tb.Th, and Tb.Sp in 8-week-old *Vhl* cKO mice and their littermate control femurs (n = 3). **(g)** Representative images of HE staining of 8-week-old *Vhl* cKO mice and their littermate control femurs. **(h)** Statistical data of Bone area/Tissue area calculated by HE staining (n = 3). **(i)** Representative

images of Goldner staining of 8-week-old *Vhl* cKO mice and their littermate control femurs. **(j)** Representative images of HIF-1α IHC staining of 8-week-old *Vhl* cKO mice and their littermate control femurs. **(k)** The average integrated density of HIF-1α IHC staining ($n = 3$). All data presented as mean ± SEM and P values were analyzed by two-tailed t-tests in a, c, d, e, f, h, and k. **$P < 0.01$, ***$P < 0.001$. All data are representative of two to three independent experiments. The data were obtained from male mice.

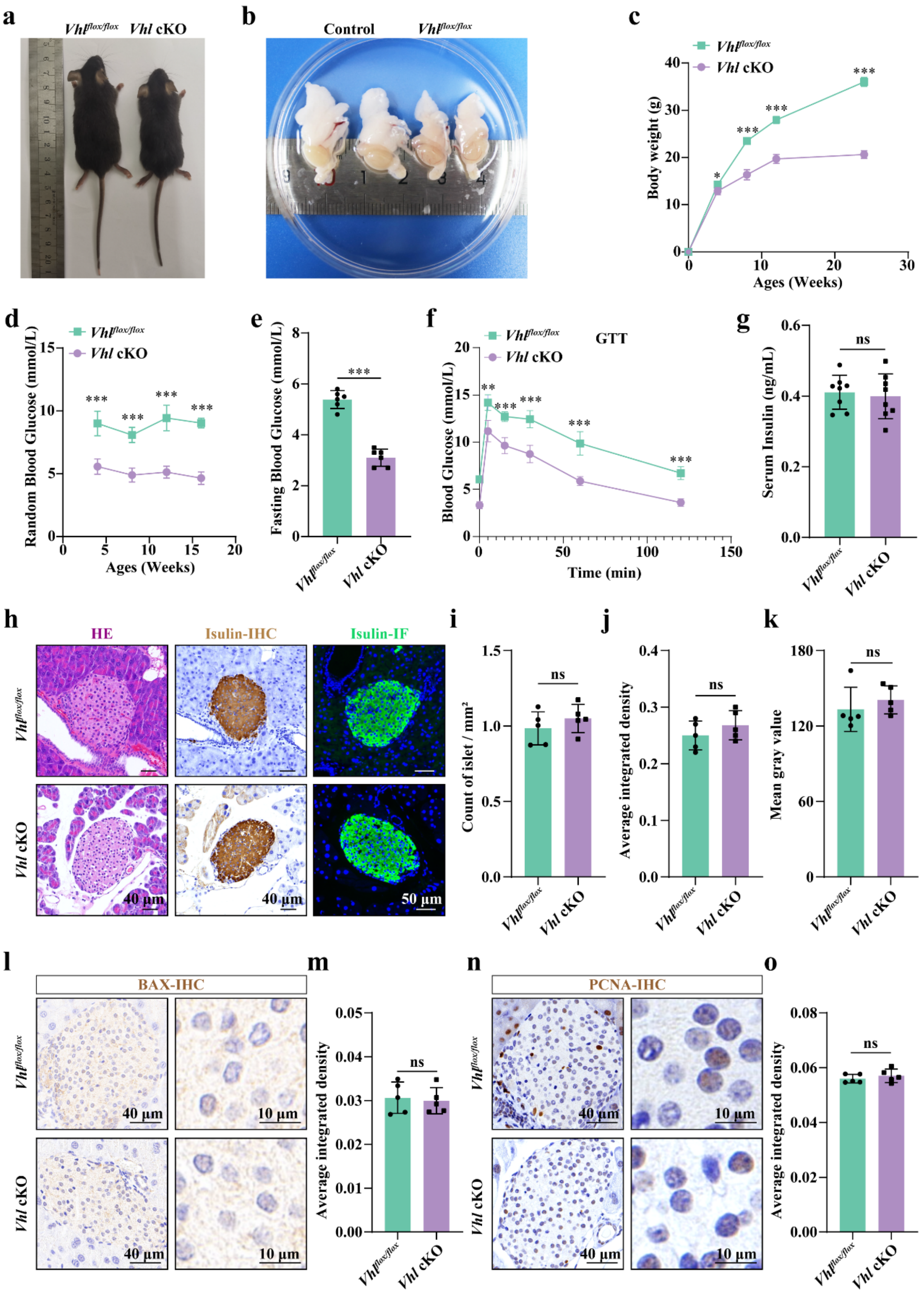

Supplementary Fig. 3. *Vhl* cKO mice are lean and show hypoglycemia and increased glucose tolerance, with no alternating changes in serum insulin levels and pancreatic β cells. (a) Photos of 8-week-old *Vhl* cKO mice and their littermate controls. **(b)** Representative images of testes and epididymal fat pads of 8-week-old *Vhl*

cKO mice and their littermate controls. **(c)** Weight changes over time of *Vhl* cKO mice and their littermate controls (n = 6). **(d)** Random blood glucose data of *Vhl* cKO mice and their littermate controls (n = 6). **(e)** Fasting blood glucose data of 8-week-old *Vhl* cKO mice and their littermate controls (n = 6). **(f)** GTT of 8-week-old *Vhl* cKO mice (n = 7) and their littermate controls (n = 5). **(g)** Serum insulin levels of 8-week-old *Vhl* cKO mice and their littermate controls under random-fed conditions (n = 8). **(h)** HE staining and insulin immunostaining of pancreatic islets of 8-week-old *Vhl* cKO mice and their littermate controls. **(i)** Statistical data of count of islet/mm^2 (n = 5). **(j)** Statistical data of average integrated density of insulin IHC (n = 5). **(k)** Statistical data of the mean gray value of insulin IF (n = 5). **(l)** BAX IHC staining of pancreatic islets of 8-week-old *Vhl* cKO mice and their littermate controls. **(m)** Statistical data of average integrated density of BAX IHC staining (n = 5). **(n)** PCNA IHC staining of pancreatic islets of 8-week-old *Vhl* cKO mice and their littermate controls. **(o)** Statistical data of average integrated density of PCNA IHC staining (n = 5). All data presented as mean ± SEM and *P* values were analyzed by two-tailed t-tests in e, g, i, j, k, m, and o, and two-way ANOVA in c, d, and f. ns, with no significant difference, $*P < 0.05$, $**P < 0.01$, $***P < 0.001$. All data are representative of two to three independent experiments. The data were obtained from male mice.

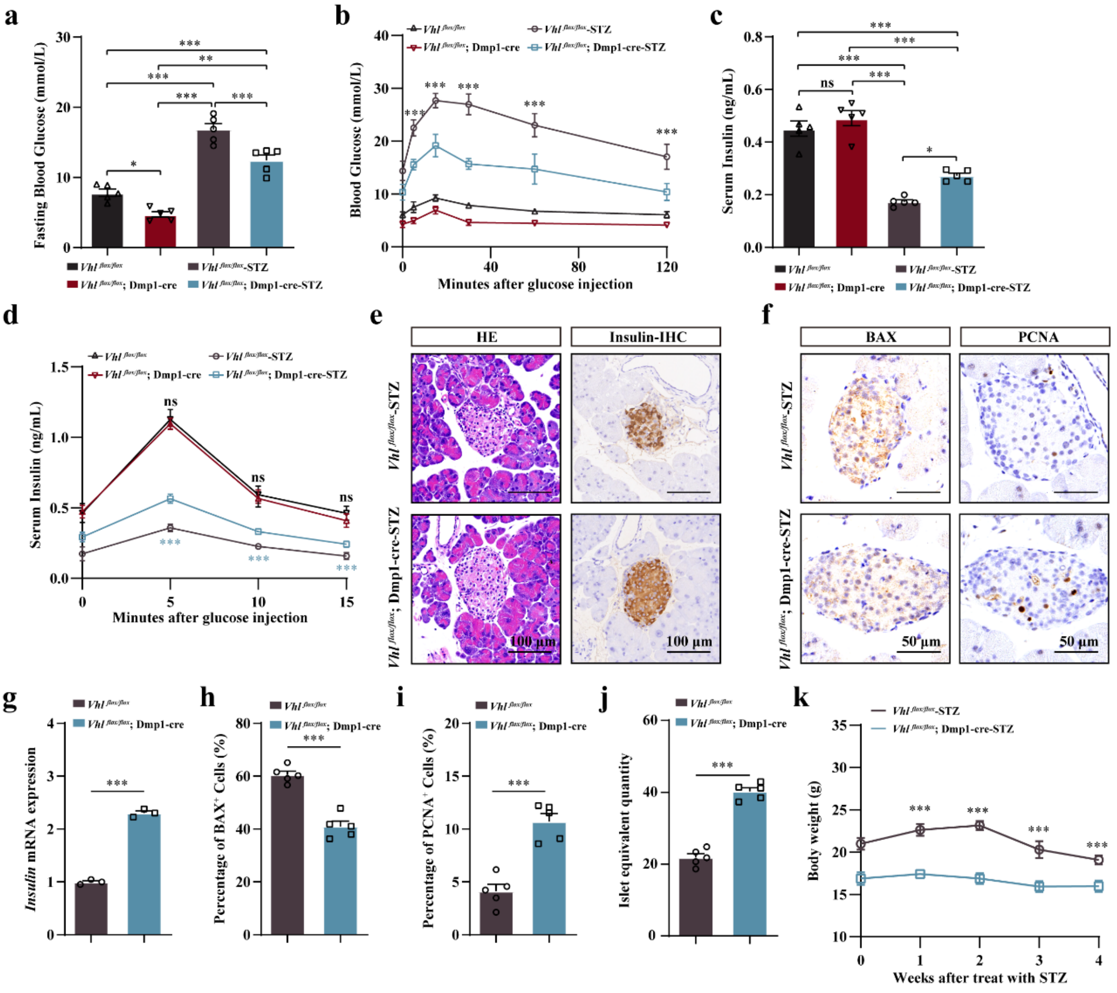

Supplementary Fig. 4. *Vhl* deletion in late osteoblasts and osteocytes via *Dmp-1*-Cre partially protects against T1DM. (a) Fasting blood glucose of *Vhl*$^{f/f}$;*Dmp-1*-Cre mice and their littermate controls treated with or without STZ (n=5). **(b)** GTT of *Vhl*$^{f/f}$;*Dmp-1*-Cre mice and their littermate controls treated with STZ (n=5). **(c)** Serum insulin level detected by ELISA. **(d)** GSIS of *Vhl*$^{f/f}$;*Dmp-1*-Cre mice and their littermate controls treated with STZ (n=5). **(e)** Representative images of pancreatic islets HE staining and insulin IHC staining of Control, *Vhl*$^{f/f}$;*Dmp-1*-Cre mice treated with STZ (n = 5). **(f, h and i)** Representative images of pancreatic islets BAX and PNCA IHC staining of Control, *Vhl*$^{f/f}$;*Dmp-1*-Cre mice treated with STZ (n = 5). **(g)** *Insulin* mRNA expression of Control, *Vhl*$^{f/f}$;*Dmp-1*-Cre mice treated with STZ (n = 5). **(j)** Islet equivalent (IEQ) calculation of pancreatic islets of Control, *Vhl*$^{f/f}$;*Dmp-1*-Cre mice treated with STZ (n = 5). **(k)** Body weight changes of Control, *Vhl*$^{f/f}$;*Dmp-1*-Cre mice treated with STZ (n = 5). All data presented as mean ± SEM and *P* values were analyzed

by two-tailed *t*-tests in g-j, one-way ANOVA in a and c, and two-way ANOVA in b, d and k. *P < 0.05, **P < 0.01, ***P < 0.001. The data were obtained from male mice.

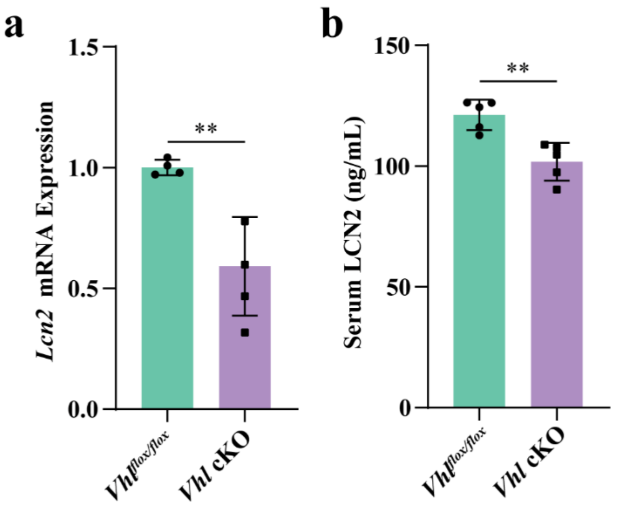

Supplementary Fig. 5. LCN2 expression in *Vhl* cKO mice and their littermate controls. (a) *Lcn2* mRNA expression in bone tissue of *Vhl* cKO mice and their littermate controls (n = 4). **(b)** Levels of LCN2 in serum of *Vhl* cKO mice and their littermate controls (n = 5). All data presented as mean ± SEM and P values were analyzed by two-tailed *t*-tests in a and b. **P < 0.01. All data are representative of two to three independent experiments. The data were obtained from male mice.

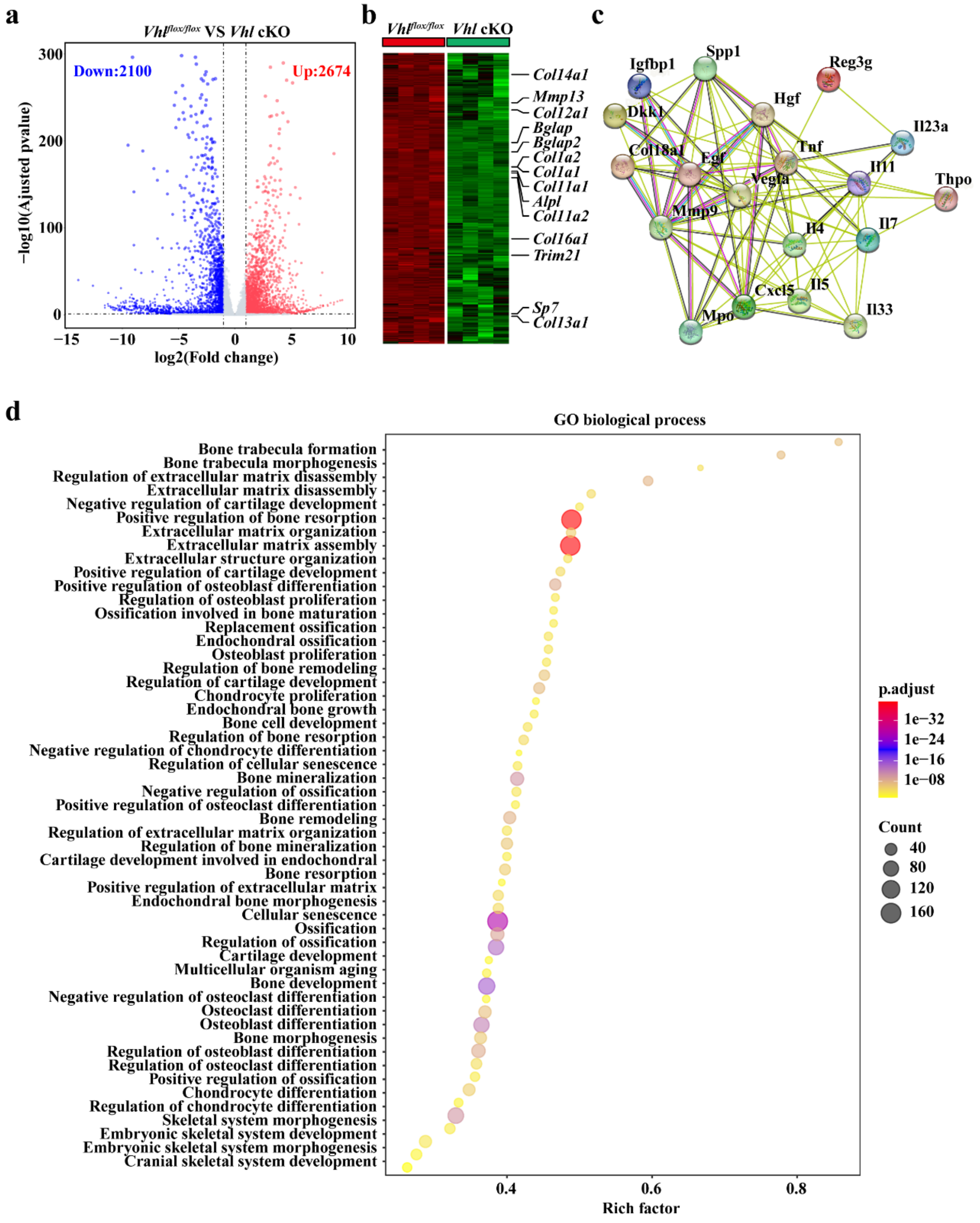

Supplementary Fig. 6. Bone tissue RNA-seq and serum cytokine bioinformatics analysis of *Vhl* cKO mice and their littermate controls. **(a)** Volcano plot of differentially expressed genes in bone tissue from *Vhl* cKO mice compared to their littermate controls. The blue and red dots represent down- and up-regulated genes, respectively. **(b)** Heat-map of down-regulated genes in bone tissue from *Vhl* cKO mice compared to their littermate controls. **(c)** Interaction plots of cytokines differentially

expressed in serum from *Vhl* cKO mice and their littermate control. **(d)** GO enrichment analysis of the signaling pathways associated with mRNAs differentially expressed between bone tissue from *Vhl* cKO mice and their littermate controls. The data were obtained from male mice.

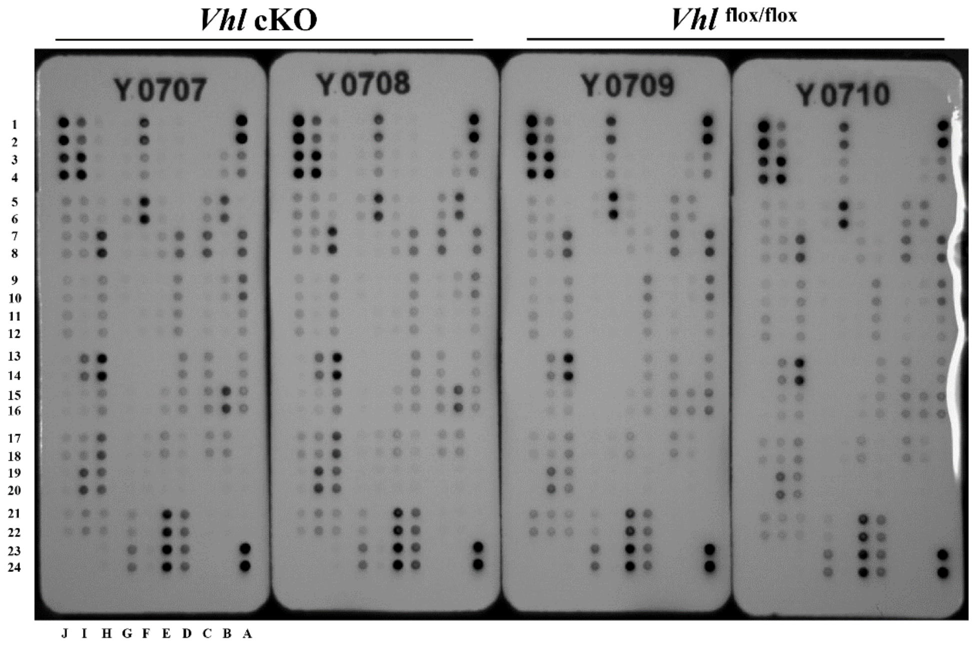

Supplementary Fig. 7. Cytokine microarray analysis of serum differentially expressed proteins in *Vhl* cKO mice and their littermate controls (*n*=4). Cytokine arrays were performed using the Proteome Profiler Mouse XL Cytokine Array Kit (Cat. # ARY028, R&D Systems). The target proteins present in the sample bind to the capture antibodies and are detected using biotinylated detection antibodies. The detection is visualized using chemiluminescent detection reagents (Bio-Rad) and imaged using a Tanon-5200 Chemiluminescent Imaging System (Tanon Science and Technology).

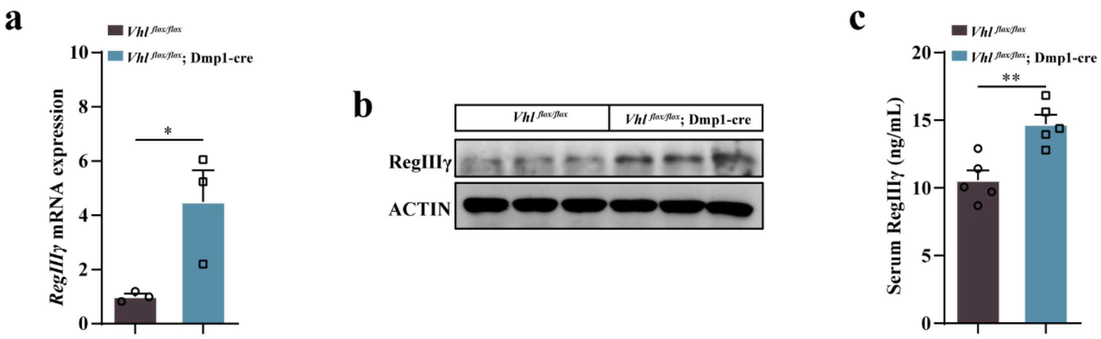

Supplementary Fig. 8. RegIIIγ expression and its circulation in *Vhl*$^{f/f}$;*Dmp-1*-Cre and *Vhl*$^{f/f}$ mice. (a and b) The mRNA and protein expression of RegIIIγ in bone tissues of *Vhl*$^{f/f}$;*Dmp-1*-Cre and *Vhl*$^{f/f}$ mice. **(c)** Serum RegIIIγ detected by ELISA. All data presented as mean ± SEM and *P* values were analyzed by two-tailed t-tests. *$P < 0.05$, **$P < 0.01$. The data were obtained from male mice.

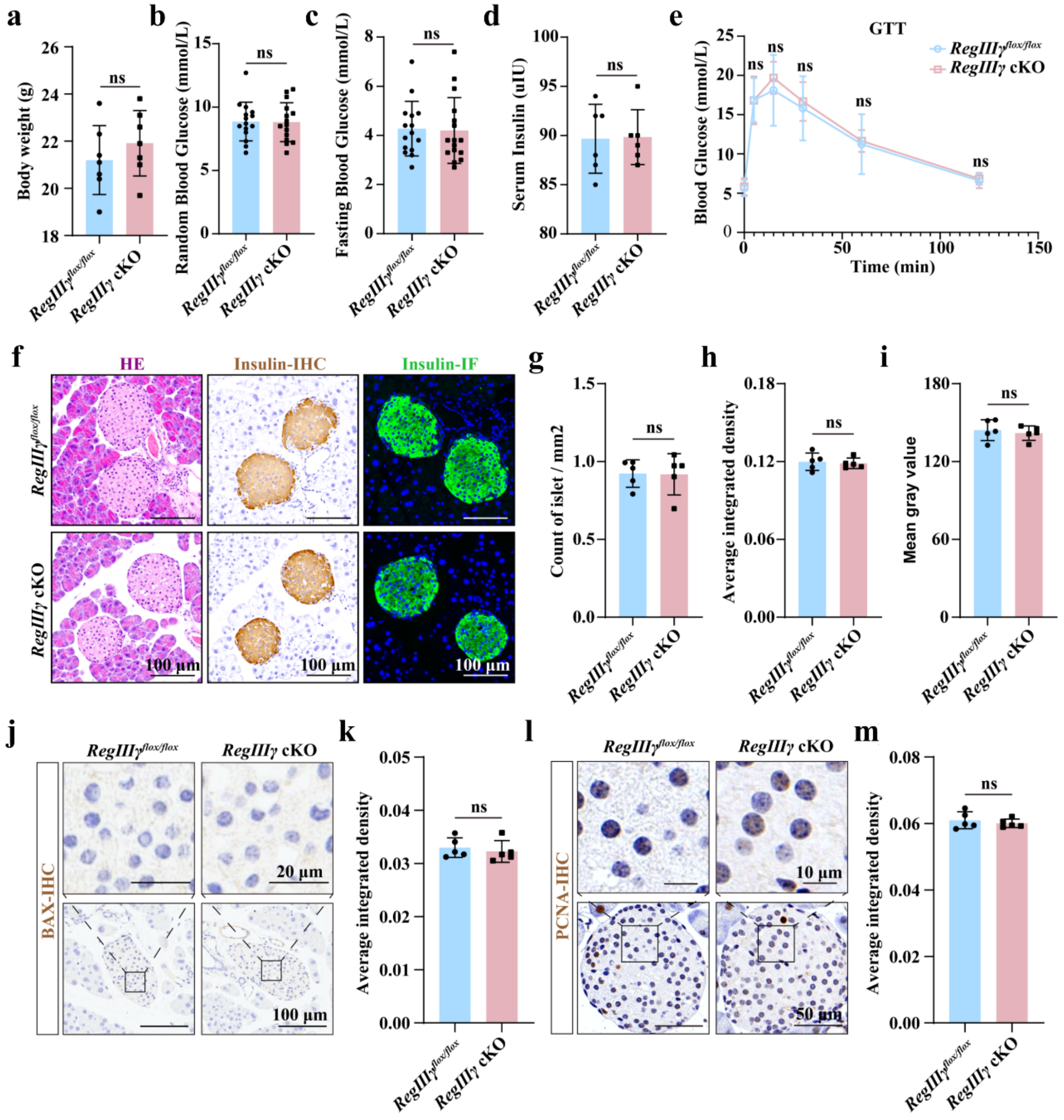

Supplementary Fig. 9. In normal physiological conditions, the body weight, serum insulin and glucose levels, GTT, and pancreatic islets of RegIIIγ cKO mice are not significantly different from the controls. (a) Body weight of 8-week-old *RegIIIγ* cKO mice and their littermate controls (*n* = 7). **(b and c)** Random and fasting blood glucose statistics of 8-week-old *RegIIIγ* cKO mice and their littermate controls (*n* = 15). **(d)** Serum insulin statistics of 8-week-old *RegIIIγ* cKO mice and their littermate controls (*n* = 6). **(e)** GTT of 8-week-old *RegIIIγ* cKO mice and their littermate controls (*n* = 6). **(f)** Representative images of pancreatic HE staining and insulin immunostaining in 8-week-old *RegIIIγ* cKO mice and their littermate controls. **(g)** Count of islet/mm^2 (*n* = 5). **(h)** The average integrated density of insulin IHC (*n* = 5). **(i)** The mean gray value

of insulin IF (n = 5). **(j)** BAX IHC staining of pancreatic islets of 8-week-old *RegIIIγ* cKO mice and their littermate controls. **(k)** The average integrated density of BAX IHC staining (n = 5). **(l)** PCNA IHC staining of pancreatic islets of 8-week-old *RegIIIγ* cKO mice and their littermate controls. **(m)** The average integrated density of PCNA IHC staining (n = 5). All data presented as mean ± SEM and P values were analyzed by two-tailed t-tests in a, b, c, d, g, h, i, k, and m, and two-way ANOVA in e. ns, with no significant difference. All data are representative of two to three independent experiments. The data were obtained from male mice.

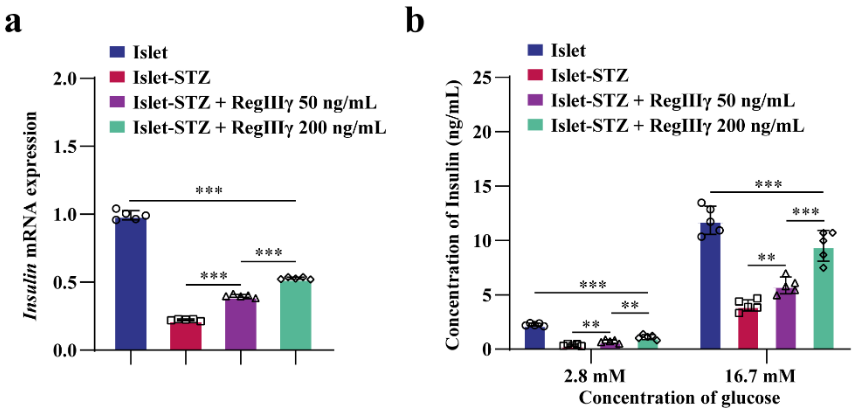

Supplementary Fig. 10. GSIS in isolated islets in response to treatment with the RegIIIγ protein. (a) Insulin mRNA in isolated islets from male mice treated with STZ and RegIIIγ. **(b)** GSIS in isolated islets treated with the STZ and RegIIIγ. (n=5). All data presented as mean ± SEM and P values were analyzed by one-way ANOVA. **P < 0.01, ***P < 0.001.

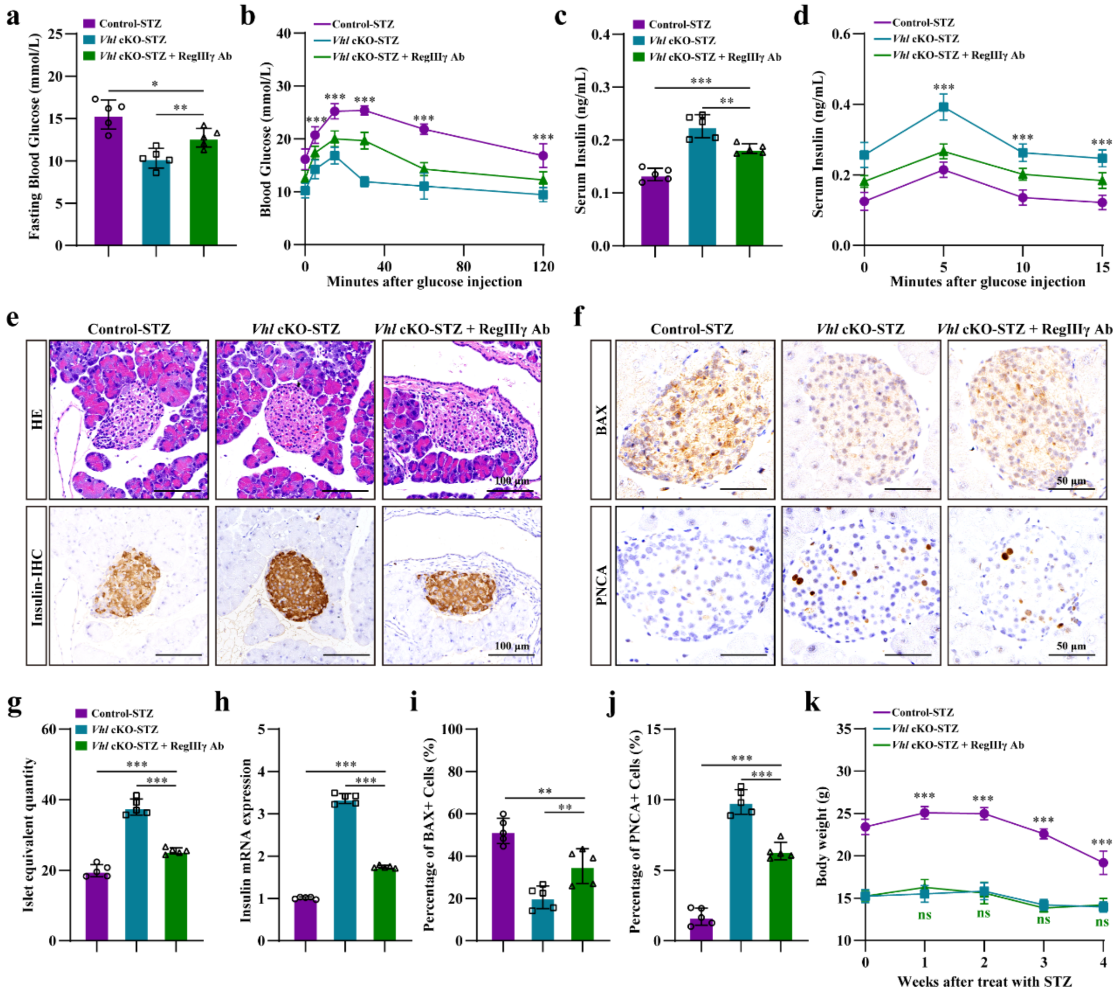

Supplementary Fig. 11. The blockade of RegIIIγ by the RegIIIγ-neutralizing antibody (Ab) impaired the protective effect of *Vhl* cKO on STZ-induced T1MD. **(a)** Fasting blood glucose in Control, *Vhl* cKO mice treated with STZ/RegIIIγ Ab (n = 5). **(b)** GTT of Control, *Vhl* cKO mice treated with STZ/RegIIIγ Ab (n = 5). **(c)** Serum insulin levels of Control, *Vhl* cKO mice treated with STZ/RegIIIγ Ab (n = 5). **(d)** Glucose-stimulated insulin secretion (GSIS) of Control, *Vhl* cKO mice treated with STZ/RegIIIγ Ab. **(e)** Representative images of pancreatic islets HE staining and insulin IHC staining of Control, *Vhl* cKO mice treated with STZ/RegIIIγ Ab (n = 5). **(f)** Representative images of pancreatic islets BAX and PNCA IHC staining of Control, *Vhl* cKO mice treated with STZ/RegIIIγ Ab (n = 5). **(g)** Islet equivalent (IEQ) calculation of pancreatic islets of Control, *Vhl* cKO mice treated with STZ/RegIIIγ Ab (n = 5). **(h)** Insulin mRNA expression of Control, *Vhl* cKO mice treated with STZ/RegIIIγ Ab (n = 5). **(i and j)** Percentage of BAX and PNCA positive β cells in (f).

(k) Body weight changes of Control, *Vhl* cKO mice treated with STZ/RegIIIγ Ab (n = 5). All data presented as mean ± SEM and *P* values were analyzed by one-way ANOVA in a, c, g, h, i, j, and two-way ANOVA in b, d and k. *$P < 0.05$, **$P < 0.01$, ***$P < 0.001$. The data were obtained from male mice.

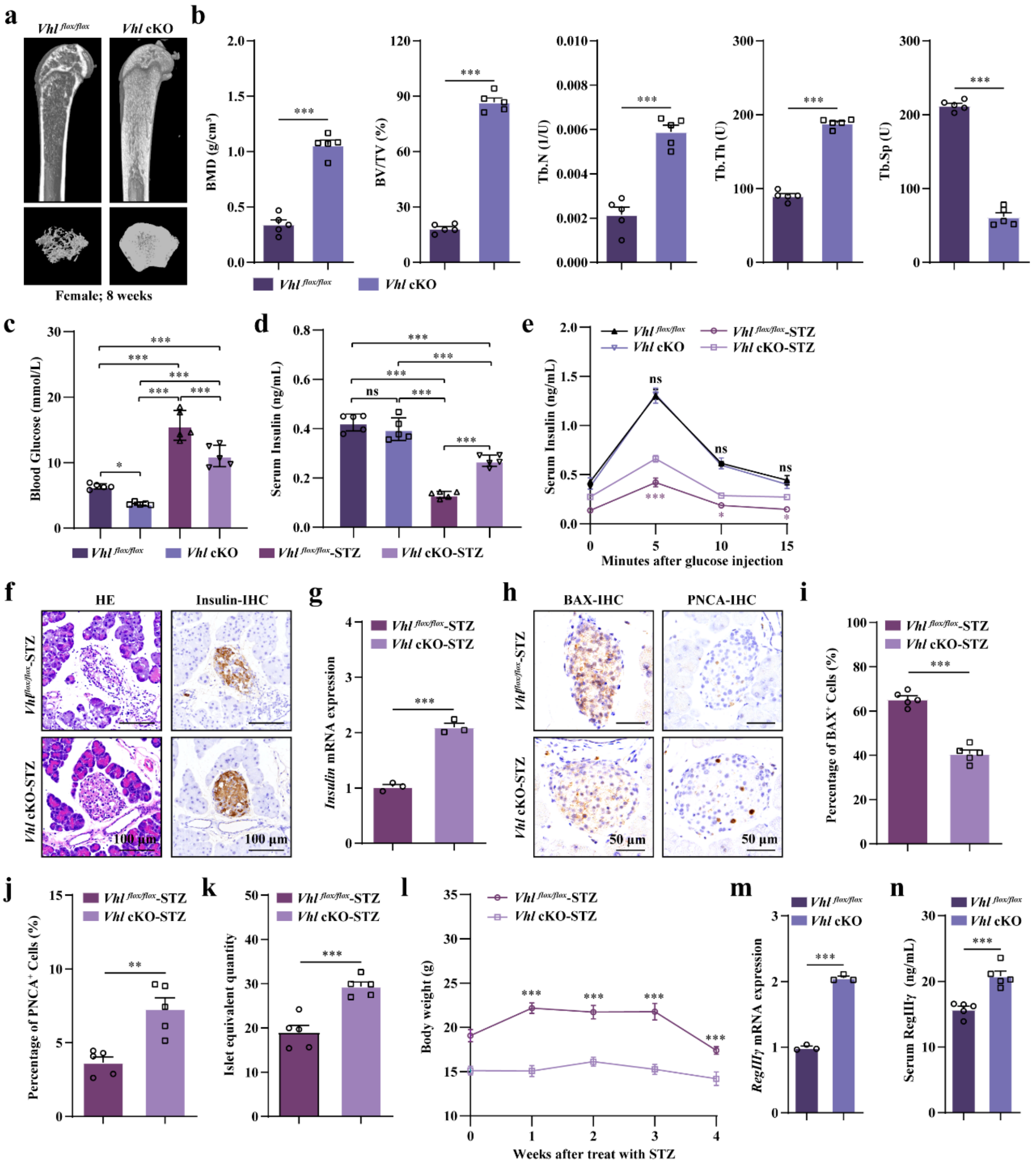

Supplementary Fig. 12. (a) Representative Micro-CT images of 12-week-old female mice with *Vhl* cKO and their littermate control femurs. **(b)** Analysis of bone trabecular parameters, including BMD, BV/TV, Tb.N, Tb.Th, and Tb.Sp in 12-week-old female

mice with *Vhl* cKO and their littermate control femurs (n = 5). **(c)** Blood glucose levels in 12-week-old female mice with *Vhl* cKO (n = 5) and their littermate controls (n = 5) after injection with STZ. **(d)** Serum insulin levels in 12-week-old female mice with *Vhl*$^{flox/flox}$ (n = 5), Vhl cKO (n = 5), STZ-treated *Vhl*$^{flox/flox}$ mice (n = 5), and STZ-treated *Vhl* cKO mice (n = 5). **(e)** *Vhl* cKO female mice demonstrate a higher ability to promote glucose-stimulated insulin secretion (GSIS) compared to the control group under STZ conditions. **(f)** Representative images of pancreatic islet HE staining and insulin immunostaining in *Vhl* cKO and control mice under STZ induction. **(g)** Insulin mRNA expression in the pancreatic islets of *Vhl* cKO and control female mice under STZ induction (n = 3). **(h, i, and j)** Islet BAX and PCNA IHC staining images of *Vhl* cKO and control female mice under STZ induction. **(k)** Calculation of IEQ (Islet Equivalent Quantity) of *Vhl* cKO and control female mice under STZ induction (n = 5). **(l)** Changes in body weight of *Vhl* cKO and control mice under STZ induction (n = 5). **(m and n)** Elevated expression of RegIIIγ in the femurs and higher levels of RegIIIγ in the serum of *Vhl* cKO female mice. All data presented as mean ± SEM and *P* values were analyzed by two-tailed *t*-tests in b, g, i, j, k, m and n, one-way ANOVA in c and d, and two-way ANOVA in e and l. *$P < 0.05$, **$P < 0.01$, ***$P < 0.001$.

Supplementary Table 1. Genotyping primers for *Vhl* flox mice

Primer name	Primer sequence（5’→3’）	PCR product size
Vhl-F	AAGAGCACGCAGCTTAGGAG	WT: 307 bp
Vhl-R	TTTCTGAGTCCTGGGGATTG	Targeted: 500 bp

Supplementary Table 2. Genotyping primers for *Ocn*-Cre and *Dmp-1*-Cre mice

Primer name	Primer sequence（5’→3’）	PCR product size
Ocn-Cre-F	CAAATAGCCCTGGCAGATTC	WT: -
Ocn-Cre-R	TGATACAAGGGACATCTTCC	Targeted: 280 bp
Dmp-1-Cre-F	TTGCCTTTCTCTCCACAGGT	WT:-

Dmp-1-Cre-R	CATGTCCATCAGGTTCTTGC	Targeted: 167 bp

Supplementary Table 3. Genotyping primers for *RegIIIγ* flox mice

Primer name	Primer sequence（5’→3’）	PCR product size
RegIIIγ-F	GCAAATCTCGCAAGTGAAAGCATAC	WT: 161 bp
RegIIIγ-R	CATGAAGTGGTAAGTGTAAGGGACC	Targeted: 262 bp

Supplementary Table 4. Primer sequences for real time-PCR

Gene		Primer sequence（5’- 3’）
β-actin	FORWARD	GGCTGTATTCCCCTCCATCG
	REVERSE	CCAGTTGGTAACAATGCCATGT
RegIIIγ	FORWARD	TTCCTGTCCTCCATGATCAAA
	REVERSE	CATCCACCTCTGTTGGGTTC
Vegfa	FORWARD	GCAGCGACAAGGCAGACTAT
	REVERSE	AACCTCCTCAAACCGTTGGC
Lcn2	FORWARD	AGCTTTACGATGTACAGCACCAT
	REVERSE	GATACCTGTGCATATTTCCCAGA
Insulin	FORWARD	CACCAGCCCTAAGTGATCCG
	REVERSE	GGCTGGGTTGAGGATAGCAA

Supplementary Table 5. Primer sequences for *RegIIIγ* promoter

	Primer sequence (5’- 3’)
FORWARD	GCGCCCGGGTTGTCAAATGTTCATGTTTCACC
REVERSE	GCAGATCTGTGTCAGGACAGAGATGAC

Supplementary Table 6. Primer sequences for CHIP-qPCR

		Primer sequence (5’- 3’)
-951 → -954	FORWARD	GAAACGTGAACTCCTTTCTGCC

	REVERSE	AGCATTAATCCATCCAGAGTCTT
-812 → -815	FORWARD	ACACAAGTGTTGGTACCATTAAAA
	REVERSE	TCCCTTAGAATGGCTTTGACG
